# Comparative Transcriptomic and Epigenomic Analyses Reveal New Regulators of Murine Brown Adipogenesis

**DOI:** 10.1371/journal.pgen.1006474

**Published:** 2016-12-06

**Authors:** Reinhard Brunmeir, Jingyi Wu, Xu Peng, Sun-Yee Kim, Sofi G. Julien, Qiongyi Zhang, Wei Xie, Feng Xu

**Affiliations:** 1 Singapore Institute for Clinical Sciences, Agency for Science, Technology and Research (A*STAR), Singapore, Republic of Singapore; 2 Center for Stem Cell Biology and Regenerative Medicine, MOE Key Laboratory of Bioinformatics, THU-PKU Center for Life Sciences, School of Life Sciences, Tsinghua University, Beijing, China; 3 Institute of Molecular and Cell Biology, A*STAR, Singapore, Republic of Singapore; ETH Zürich, SWITZERLAND

## Abstract

Increasing energy expenditure through brown adipocyte recruitment is a promising approach to combat obesity. We report here the comprehensive profiling of the epigenome and transcriptome throughout the lineage commitment and differentiation of C3H10T1/2 mesenchymal stem cell line into brown adipocytes. Through direct comparison to datasets from differentiating white adipocytes, we systematically identify stage- and lineage-specific coding genes, lncRNAs and microRNAs. Utilizing chromatin state maps, we also define stage- and lineage-specific enhancers, including super-enhancers, and their associated transcription factor binding motifs and genes. Through these analyses, we found that in brown adipocytes, brown lineage-specific genes are pre-marked by both H3K4me1 and H3K27me3, and the removal of H3K27me3 at the late stage is necessary but not sufficient to promote brown gene expression, while the pre-deposition of H3K4me1 plays an essential role in poising the brown genes for expression in mature brown cells. Moreover, we identify SOX13 as part of a p38 MAPK dependent transcriptional response mediating early brown cell lineage commitment. We also identify and subsequently validate PIM1, SIX1 and RREB1 as novel regulators promoting brown adipogenesis. Finally, we show that SIX1 binds to adipogenic and brown marker genes and interacts with C/EBPα, C/EBPβ and EBF2, suggesting their functional cooperation during adipogenesis.

## Author Summary

Obesity and its related metabolic diseases are growing problems worldwide. Brown adipose tissue (BAT) with its capability of burning off fat to generate heat is now at the center of research interest as target of therapeutic intervention for obesity treatment. In order to get a better understanding of the molecular mechanisms and transcriptional programs underlying brown adipocyte differentiation, we profiled the epigenomic and transcriptomic changes during brown adipogenesis and performed a comparative analysis against white adipogenesis using bioinformatic tools. We identified several novel factors involved in brown adipocyte differentiation and showed that the kinase PIM1 and the transcription factors SIX1, SOX13 and RREB1 positively regulate differentiation. Finally we also provide a genome-wide map of SIX1 binding in mature brown adipocytes.

## Introduction

Obesity and its associated metabolic complications such as diabetes are increasingly responsible for significant economic and social burdens in many countries worldwide. Physiologically, obesity develops when energy intake exceeds energy expenditure, and the current treatments of obesity have been primarily focused on reducing energy intake. Unfortunately, these measures were largely inefficient in maintaining long-term weight loss [1]. The recent discovery of thermogenic adipocytes [2–5] capable of burning fat in adult humans has provided an exciting new therapeutic approach for the treatment or prevention of obesity by increasing energy expenditure [6].

Fat cells are derived from multipotent mesenchymal stem cells (MSCs), which can give rise to muscle, adipose, bone, or cartilage cells when given appropriate environmental cues. These cells can be broadly divided into fat storage cells, such as white adipocytes (WA); and fat burning cells, which include classical and inducible brown adipocytes (BA) (also known as beige or brite adipocytes) [7]. Brown cells contain high density of mitochondria and dissipate chemical energy as heat through the action of the mitochondrial protein UCP1 (uncoupling protein 1). It is evident that increased activity of thermogenic brown cells has beneficial effects on whole body metabolic homeostasis [3, 5, 8] and various environmental cues such as cold exposure and chemical activation of the β-adrenergic pathway can significantly up-regulate BAT activity [2, 9]. Over the last couple of years, a number of protein factors as well as long non-coding RNAs (lncRNAs) and microRNAs have been identified as regulators in this process. For example, the members of the bone morphogenetic protein (BMP) family, the PPARγ co-factor PGC1α, the transcription factors (TFs) PRDM16, EBF2, KLF11, the protein deacetylase SIRT1, the secreted factors IRISIN and FGF21 as well as lncRNAs Blnc1, lncBATE1, and microRNAs miR193/365 have been shown to be essential for thermogenic fat cell recruitment [6, 10–13].

To promote thermogenic adipocyte recruitment, it is necessary to have a fundamental understanding of the gene regulation networks that control brown and white adipogenesis and identify the key differences between these two morphologically similar but functionally distinct cell types. Gene regulation networks are composed of *cis*-regulatory elements and *trans*-regulatory protein factors. In response to environmental stimuli, *trans*-factors bind to *cis*-elements such as enhancers or silencers to modulate gene expression. Given the large size and complexity of mammalian genomes, it has been difficult to systematically identify *cis*-regulatory elements at the genome-wide level. The recent discovery of signature histone modifications for these *cis*-elements (eg. H3K27 acetylation for active enhancers) and the advance in massive parallel DNA sequencing facilitated to comprehensively define these elements. In addition to typical enhancers, a group of so-called super-enhancers was discovered recently. These super-enhancers are large enhancer clusters containing high-density transcription factor binding and are associated with cell type specific genes [14]. They are also stronger in terms of gene activation ability and play key roles in controlling cell identity in mammals.

*Trans*-regulatory factors are mainly identified through differential gene expression and genetic analyses. For example, the prominent adipogenic factors PPARγ and C/EBPα are strongly up-regulated during the course of adipogenesis. Recently some of the TFs were also discovered by analyzing the TF binding motif in cell type specific enhancer elements. Through this approach, PLZF and SRF were identified as anti-adipogenic factors [15] while EBF2 was identified as an activator for brown adipogenesis [16]. Finally, the presence of a super-enhancer at the proximity of *Klf11* gene in human adipocytes led to its identification as a browning factor [10].

Although previous studies have looked at individual histone modification (H3K27ac) occupancy in mature brite cells [10] and brown adipose tissue [17], a comprehensive profiling including other important chromatin marks during brown adipogenesis is still lacking. For example, H3K4me3 marks the promoters of actively transcribed genes [18]; H3K4me1 and H3K9ac are found at active/poised enhancers/promoters [19]; In contrast, H3K27me3 is a repressive mark and enriches at polycomb-repressed loci [20]. This information is important for describing epigenomic landscapes and is often required for downstream bioinformatic analyses. For instance, active enhancer regions are defined by the presence of H3K27ac and the absence of H3K4me3 [21], and bivalent domains are enriched by both H3K4me3 and H3K27me3 [22].

Extensive studies have been carried out to characterize the dynamic chromatin regulation of WA differentiation [15], but the global chromatin landscape for BA lineage specification and differentiation is far from complete. Especially, the epigenomic transition state that is not observed in cells either at the beginning or end of adipogenesis and which is essential for the transmission of adipogenic signals [23], has not been analyzed in detail in the brown lineage. In this study, we adapted a protocol to efficiently differentiate the murine MSC line C3H10T1/2 from multipotent precursors into mature BAs via pre-treatment with BMP7. The secreted factor BMP7 has been shown to be essential for brown adipogenesis *in vitro* and *in vivo* and important for BA lineage specification [24]. We collected samples at five important time points, representing specific developmental stages ranging from (1) multipotent mesenchymal stem cells, (2) committed brown preadipocytes, cells during (3) early and (4) late intermediary transition states, to (5) mature BAs. Using these samples, we generated epigenomic maps for a number of key histone modifications, PPARγ binding, together with corresponding gene expression profiles including mRNAs, lncRNAs and microRNAs. To identify regulators specific for brown adipogenesis, we compared the BA dataset to the results from white adipogenesis and performed analysis of: (1) stage- and lineage-specific gene expression; (2) stage- and lineage-specific enhancer enriched TF binding motifs; (3) lineage-specific super-enhancers; (4) BMP7 responsive genes. Through these analyses we not only re-discovered most established regulators of brown adipogenesis, but also identified a number of novel putative activators of BA differentiation including the kinase PIM1, and three TFs, SIX1, RREB1 and SOX13. Via gain- and loss-of-function analyses, we validated that these factors are indeed essential for BA lineage commitment or differentiation. Finally, we mapped and analyzed the genome-wide binding of SIX1 in BAs using ChIP-seq. We found that its binding sites are enriched for C/EBP and EBF motifs and co-immunoprecipitation (co-IP) experiments confirmed that SIX1 physically interacts with C/EBPα, C/EBPβ and EBF2, suggesting their cooperation during BA differentiation. Moreover, through analysis of the chromatin dynamics at brown lineage-specific genes, we found these genes to be pre-marked by both H3K4me1 and H3K27me3, and H3K27 demethylation at the late stage was not sufficient to promote their expression, indicating an essential role for H3K4me1 in poising brown genes for expression. In summary, we provide a comprehensive reference map for the dynamic epigenome and transcriptome during BA differentiation, propose a conceptual model of brown gene regulation, and also show that comparative transcriptomic and epigenomic analysis is a powerful tool for the discovery of novel regulators, resulting in the identification of four activators of BA differentiation in this study.

## Results

### Comprehensive profiling of the transcriptome and epigenome during murine brown adipogenesis

**Fig 1:**
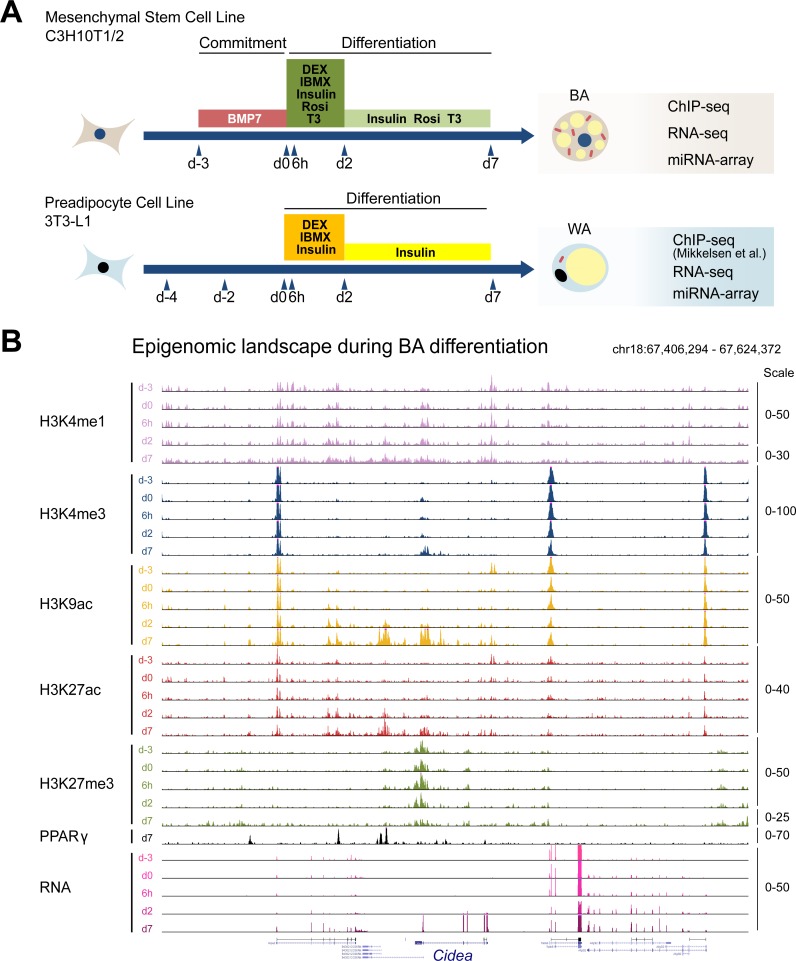
Generation of comprehensive epigenomic and transcriptomic reference maps during brown adipogenesis. (A) Schematic of the adipogenesis procedures for BA (commitment and differentiation) and WA (differentiation only). (B) Snapshot of the genomic region surrounding the *Cidea* gene in the UCSC Genome Browser featuring a panel of chromatin marks, PPARγ binding, and mRNA levels during BA differentiation. The scale represents the normalized reads counts. For the H3K4me1 and H3K27me3 d7 tracks, the scale was adjusted to account for lower total ChIP-seq signals. For other loci see S3 Fig.

To examine the molecular control of cell fate transitioning from uncommitted progenitor cells to BAs, we used C3H10T1/2 MSCs and differentiated them into BAs, following a previously established protocol [24], where the multipotent progenitors were first committed to the brown lineage by BMP7 treatment before differentiation was triggered using a chemical cocktail (Fig 1A). In addition, the well-established WA differentiation model 3T3-L1 was included in this study for comparison of events during differentiation. We first confirmed the efficiency and specificity of our differentiation systems by visual inspection of cell morphology, by qRT-PCR, and Western blot analyses of lineage marker gene expression (S1 Fig). The differentiation process for both lineages was highly efficient, as virtually all cells accumulated lipid droplets by day 7 of differentiation (S1A and S1B Fig). As expected, *Pparg2*, the master regulator of adipocyte differentiation, and other adipogenic marker genes such as *Fabp4*, *CD36*, *Lpl*, *Adipoq* and *Cebpa* were strongly up-regulated in both lineages in response to differentiation signals. In contrast, BA marker genes including *Ucp1*, *Cidea*, *Elovl3*, *Ppara* and *Prdm16* were activated only in mature BAs, and the mitochondrial marker genes *Cox7a1* and *Cox8b* were expressed much higher in mature BAs (S1C and S1D Fig). Corresponding expression patterns were also detected at the protein levels for PPARγ, UCP1, PPARα and CIDEA (S1E Fig). These data indicated that our differentiation processes were specific and efficient.

Next we profiled the transcriptome and epigenome during murine brown adipogenesis at five key time points: (1) day -3 (d-3, uncommitted progenitors); (2) day 0 (d0, end of brown lineage commitment by BMP7 treatment); (3) 6 hours (6h, end of epigenomic transition [23]); (4) day 2 (d2, early BA differentiation) and (5) day 7 (d7, mature BAs) (Fig 1A). For transcriptome profiling, we used RNA-seq for mRNAs and lncRNAs, and an array-based method for microRNAs. To validate our transcriptomic analysis during brown adipogenesis, a second replicate of the RNA-seq experiment was performed and the results indicated that the data were highly reproducible (S1 Table). In parallel, we also profiled the transcriptome using RNA-seq for mRNAs and lncRNAs, and microarray for microRNAs during 3T3-L1 WA differentiation. When compared with the transcriptomes of mouse adipose tissues, we found that our *in vitro* BA and WA systems are closely related to their corresponding *in vivo* tissues (S1 Table). To complement the analysis of transcriptional changes during brown adipogenesis, we also performed a comprehensive profiling of the dynamically changing chromatin landscape during BA differentiation by ChIP-seq. In this effort, we mapped a number of key chromatin marks including H3K4me1, H3K4me3, H3K9ac, H3K27ac and H3K27me3 during BA differentiation. We also performed replicates at two key time points (d0 and d7) for all histone marks and the results showed that our ChIP-seq data were highly reproducible (see S1 Table and S2A–S2C Fig). In addition, we profiled PPARγ binding using ChIP-seq in mature BAs where it is highly expressed. Examples of the epigenomic and transcriptomic landscapes as well as PPARγ binding during BA differentiation at the brown selective genes *Cidea*, *Ucp1* and *Ppara* are shown in Fig 1B and S3 Fig. A corresponding epigenomic dataset for WA differentiation has been generated previously [15] and was used for subsequent comparative analyses. Prior to further analysis we validated our ChIP-seq datasets by examining the correlations between gene expression and various histone modifications. As expected, we found that highly transcribed genes were marked by active chromatin marks (H3K9ac, H3K4me1, H3K4me3, and H3K27ac) but not the repressive mark H3K27me3 at their promoters (S1 Table and S2D Fig). Together, these datasets constituted comprehensive reference maps of the epigenome and transcriptome for both BA and WA differentiation.

### Stage- and lineage-specific gene expression during BA and WA differentiation

**Fig 2:**
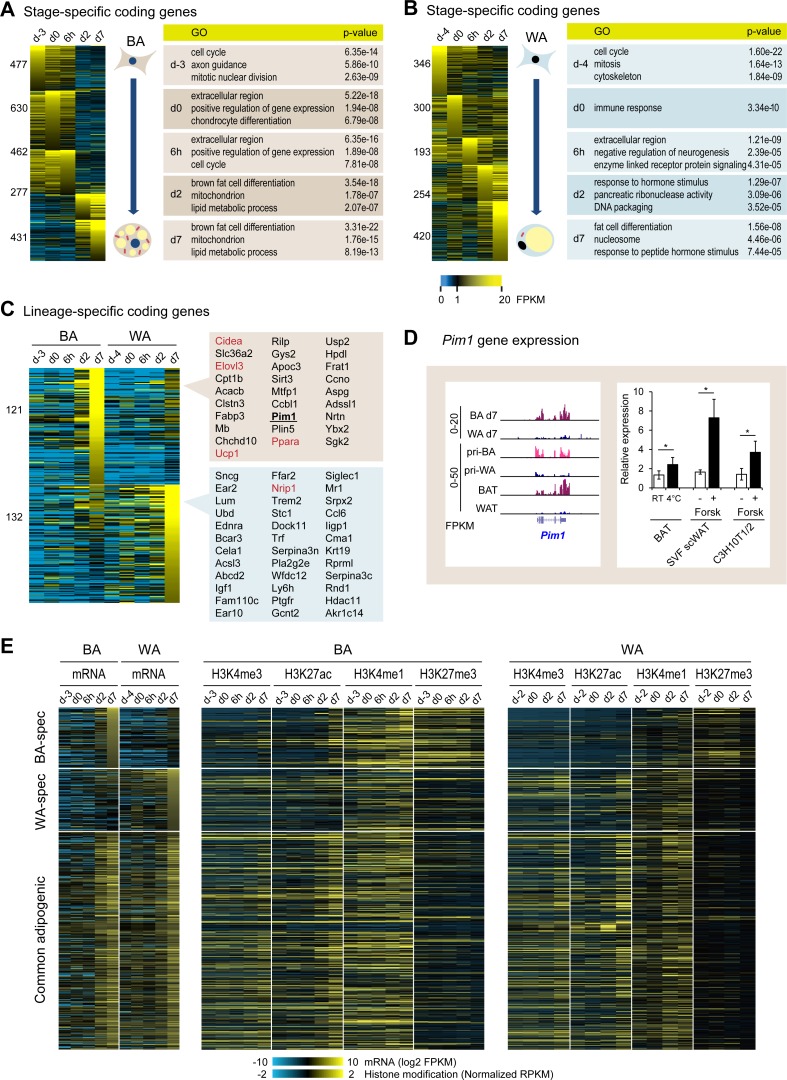
Transcriptomic analysis of BA and WA differentiation. (A) and (B) Differentiation stage-specifically expressed coding genes during BA and WA differentiation (FPKM > 5). The tables show the top 3 (non-redundant) enriched GO categories at individual stages. See S2 Table for the full list. (C) BA and WA lineage-specific coding genes. The list highlights genes showing consistent lineage specific expression between this dataset and primary cells as well as *in vivo* tissues. Several established lineage markers are highlighted in red. (D) *Pim1* gene expression. Left panel: expression pattern of the *Pim1* kinase gene determined via RNA-seq in mature BA and WA, differentiated primary SVF cells derived from BAT (pri-BA) and WAT (pri-WA), as well as brown adipose tissue (BAT) and white adipose tissue (WAT). Right panel: *Pim1* expression in BAT tissue isolated from male C57BL/6 mice (BAT); in SVF cells isolated from the posterior subcutaneous WAT and differentiated into adipocytes (SVF scWAT); in C3H10T1/2 cells differentiation into BAs (C3H10T1/2). Mice were housed either at RT or 4°C for 7days before isolation of BAT tissue. n = 3 for each group, error bars indicate standard deviation. p-values (paired student’s t-test): * < 0.05. Cells were treated either with 10μM Forskolin or DMSO for 3h before harvesting. Graphs represent the average of three independent experiments, error bars indicate standard deviation. p-values (paired student’s t-test): * <0.05. (E) Heatmaps showing the expression patterns of BA-specific, WA-specific and common adipogenic genes, as well as the status of chromatin modifications at corresponding promoters throughout the differentiation of BAs and WAs.

We first focused on genes that were dynamically regulated at different stages during adipogenesis, following the rationale that differentiation stage-specific expression mirrors functional roles for those genes. To this end, we systematically examined coding genes, lncRNA genes, and microRNA genes (Fig 2, S4 and S5 Figs, S2 Table). Using an entropy based method (See “Materials and Methods” section for details), we identified a total of 2277 (BA) and 1513 (WA) differentiation stage-specific coding genes (FPKM>5), which were 26.2% and 16.0% of the expressed genes in the respective lineages. The higher number and proportion of genes with dynamic expression in BA are in agreement with the requirement of executing additional gene programs to commit MSCs into the adipogenic lineage before differentiation. We noted a clear separation into five stages with little overlap of stage-specifically expressed genes in WAs indicating a strictly step-wise differentiation process. Specifically, 3T3-L1 cells are at the proliferation stage at d-4; at d0, these cells have been under growth arrest for 2 days [25]; after the adipogenic induction, the arrested cells re-enter the cell cycle and undergo an epigenomic transition stage at 6h [23]; while at d2, these cells are arrested again and start to differentiate [25]; finally at d7, 3T3-L1 cells are fully differentiated into mature adipocytes. In contrast, in BAs we observed a more substantial transition in gene expression between 6h and d2, whereas the time points before (d-3, d0, 6h), and after (d2, d7) showed a certain overlap of gene expression. This differentiation stage-specific gene expression pattern is likely derived from the fact that C3H10T1/2 cells continuously proliferate from d-3 to 6h without the contact inhibition and growth arrest stages observed in 3T3-L1 cells, and these cells start to accumulate fat earlier than 3T3-L1 cells (S1A and S1B Fig) at d2. To analyze our observations more systematically, we performed gene ontology (GO) analysis of stage-specific genes. The top category of enriched genes before differentiation (d-3/d-4) was “cell cycle” in both lineages, whereas the transient enrichment of “chondrocyte differentiation” was only found in BA after lineage commitment. Strikingly, the same enriched gene categories topped the list in BA at d2 and d7, i.e. “brown fat cell differentiation”, “mitochondrion”, and “lipid metabolic process”; but in WAs “fat cell differentiation” tops the list not before d7. This correlates with a well advanced differentiation status and accumulation of lipid droplets by d2 in BA, but not WA (see S1A and S1B Fig), and may explain similar gene expression pattern between d2 and d7 in BA.

We also examined lncRNA genes that are dynamically regulated during adipogenesis (S4B Fig and S2 Table). Using the NONCODE database [26], we found in total 1985 and 2796 expressed putative lncRNA genes during BA and WA differentiation (FPKM>0.5), respectively. Among them, 857 (BA) and 1135 (WA) lncRNAs showed a stage-specific expression pattern, which were 43.2% and 40.6% of the expressed lncRNAs, respectively. The proportions of stage-specific lncRNAs in BA and WA were therefore considerably higher than the ones for mRNAs which were 26.2% and 16.0%, suggesting lncRNA genes were regulated more dynamically during adipogenesis than coding genes. This trend was maintained even when the comparison was limited to lowly expressed mRNAs with similar expression levels as lncRNAs, which turned out to be the least dynamic between stages (15.5% and 10.8%). In addition, we also found a previously identified lncRNA (Blnc1) that drives thermogenic adipocyte differentiation [11] to be specifically expressed in mature BAs (S4B Fig). Finally, we profiled microRNA gene expression along the same process, leading to the identification of known general adipogenic microRNAs (e.g. miR-378), brown lineage-specific microRNAs (miR-193), as well as several microRNAs not implicated in adipogenesis so far (S5 Fig and S2 Table).

To identify lineage-specific genes, we compared the 431 genes that were stage-specifically expressed in mature BAs (d7, Fig 2A) to those 420 genes that were specifically expressed in mature WAs (d7, Fig 2B). Among them, we found that 121 genes were robustly expressed specifically in mature BAs but not WAs, and 132 genes were only expressed in mature WAs (Fig 2C). To further compile a list of putative lineage-specific markers for BA and WA, we selected the genes showing a similar lineage-specific expression pattern both in mouse BAT/WAT tissues and primary brown/white adipocytes, according to previously published data [27] (See S4A Fig for examples). The list for BA-specific genes contained a number of classic BA markers, such as *Ucp1*, *Elovl3*, *Ppara* and *Cidea*. In addition, *Slc36a2* (also known as *Pat2*), recently described as a brown/beige-specific surface marker [28]; *Cpt1b*, the rate-controlling enzyme for long-chain fatty acid β-oxidation and several other mitochondrial protein genes (*Chcd10*, *Sirt3*, *Mtfp1*, *Aspg* and *Adssl1*) were also identified in this list. This observation is consistent with increased number and activity of mitochondria in BAs. Of note, we also found the gene encoding the kinase PIM1 that was specifically expressed in BAs, primary BAs, and BAT. In addition, we also noticed increased *Pim1* expression upon cold exposure and chemical activation of the β-adrenergic pathway (Fig 2D). PIM1 belongs to a group of constitutively active serine/threonine kinases and has been implicated in a number of biological functions such as apoptosis and cell cycle regulation. Recent reports suggested that PIM1 might play a role in cellular metabolism by modulating the phosphorylation status of AKT [29] and AMPK [30]. Based on this evidence, we selected PIM1 for further functional analysis as a potential regulator of BA differentiation (detailed below). For WAs, only few markers were established before, of which we rediscovered *Nrip1* (also known as *Rip140*), a co-repressor that plays an important role in repressing a number of brown selective genes [31]. Another WA-specific gene, *Trem2*, was recently shown to enhance adipogenesis, promote glucose and insulin resistance, and diminish energy expenditure. Several other genes identified, such as the nuclear receptor *Ear2* (*Nr2f6*), the ubiquitin gene *Ubd* (*Fat10*), the free fatty acid receptor *Ffar2*, and the insulin-like growth factor *Igf1*, were shown to be involved in metabolism without a clear role in white adipogenic differentiation. Finally, we also provide a list of lineage-specific lncRNAs (S2 Table, see S4B Fig for examples). Together, this transcriptomic dataset provides a valuable resource for the identification and further characterization of novel regulators for brown and white adipogenesis.

To ask how the lineage-specific and the commonly expressed genes in BA and WA are regulated at the chromatin level, we examined the histone modification dynamics at the gene promoters throughout both BA and WA differentiation. As expected, chromatin marks for active promoters such as H3K4me3 and H3K27ac correlate well with gene activity at the promoter regions in both lineages. Given that a recent study [32] suggested that the removal of H3K27me3 is required for brown gene expression, it was not surprising to observe a decrease of this mark at the promoters of brown specific genes in BA (Fig 2E). Surprisingly, in WA, where these BA selective genes were not expressed, we also found a significant decrease in H3K27me3 at their promoters (Fig 2E and S6A Fig), suggesting that the removal of H3K27me3 is not sufficient to induce the expression of these brown specific genes. Intriguingly, in contrast to WA, we found significantly higher levels of H3K4me1 at the promoters of BA specific genes throughout BA differentiation (Fig 2E and S6B Fig). This observation suggested that the pre-deposition of H3K4me1 at the early stages of brown adipogenesis was required for efficient expression of these genes at the late stage, while the removal of H3K27me3 was necessary but not sufficient to promote brown gene expression. In parallel, we found that general adipogenic genes are only marked by H3K4me1 but not H3K27me3 during both brown and white adipogenesis, suggesting their activation does not involve H3K27 demethylation.

### TF binding motif analysis in stage-specific enhancers during BA and WA differentiation

**Fig 3:**
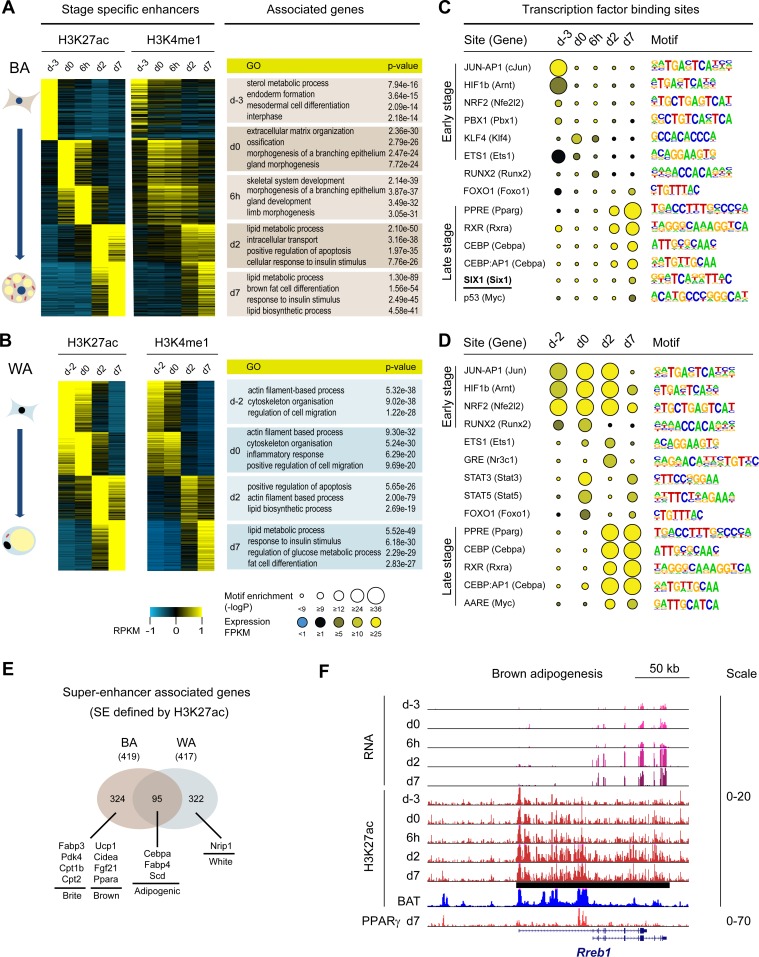
TF binding motif analysis of stage specific enhancers and identification of super-enhancer associated genes during brown adipogenesis. (A) and (B) Differentiation stage-specific enhancers (defined as H3K27ac positive and H3K4me3 negative regions) are also marked by H3K4me1 and associate with genes reflecting their developmental stages. (C) and (D) TF binding motif analysis of stage specific enhancers with HOMER. “Site” indicates the bound TF(s), “Gene” indicates the corresponding gene coding for (one of) the TF(s). Expression of genes was determined using RNA-seq data. Sites with enriched motifs and correspondingly expressed genes were shown in the list. In differentiated BAs and WAs, the binding motifs for PPARγ, RXR and C/EBP were enriched, whereas the SIX1 motif was only enriched in BAs at late stage. (E) Genes associated with super-enhancers (SEs) in mature BAs and WAs. A subset of SE associated genes is shared between BAs and WAs, including established adipogenic markers such as *Cebpa* and *Fabp4*, whereas known brown and brite selective genes are specifically associated with SEs in BAs. (F) The *Rreb1* gene was significantly up-regulated during brown adipogenesis and is associated with a SE in BAs.

Enhancers are *cis*-regulatory elements that can activate gene expression over distance. It has been shown that enhancers are highly dynamic and play an important role in cell fate transitions [21]. To examine the dynamic regulation of enhancers during BA and WA differentiation, we identified stage-specific enhancers based on the enrichment of H3K27ac, a histone mark for active enhancers and promoters [33], and the lack of H3K4me3, a histone mark present at active promoters. We employed a similar entropy-based method as for the identification of stage-specific genes and found 24,002 and 13,429 genomic loci acting as putative stage-specific enhancers throughout BA and WA differentiation (Fig 3A and 3B). Again the higher number of dynamic enhancers in BA is in agreement with the additional commitment step in the differentiation of MSCs into the brown lineage, as compared to white adipogenesis starting from committed 3T3-L1 preadipocytes. Moreover, we observed the emergence of a distinct group of stage-specific enhancers after BMP7 treatment (compare d-3 to d0, Fig 3A), which suggested an epigenomic reprogramming during the process of brown lineage commitment. Consistent with a previous report [23], we also observed an epigenomic transition as evidenced by the formation of a new group of stage-specific enhancers within 6 hours after adipogenic induction (compare d0 to 6h, Fig 3A), while from d2 to d7, there are less changes as compared to the earlier stages (Fig 3A). During white adipogenesis, the stage-specific enhancers at the early (d-2, d0) and late stages (d2, d7) show a certain overlap, while between d0 and d2, there is a relatively more drastic transition in enhancer formation. This pattern can be explained as at d-2 and d0, 3T3-L1 cells are under the growth arrest state [25]; after adipogenic induction, these cells go through clonal expansion between d0 and d2 [25]; beyond d2, the epigenomic reprogramming has been completed and the cells start to accumulate fat and subsequently enter the end differentiation stage at d7. To validate our analysis, we surveyed the levels of H3K4me1, another enhancer-associated epigenetic mark, at those loci and found a high concurrence between H3K27ac and H3K4me1 (Fig 3A and 3B and S1 Table). Analysis of the genes present in the proximity of stage-specific enhancers showed that they fall into similar GO categories as the stage-specific genes analyzed earlier. This observation suggests that the stage-specific gene expression was likely regulated by the stage-specific enhancers, further confirming the role of enhancers in cell fate transitions [21].

Enhancers often serve as hubs for TFs. To identify potential TFs that bind to these stage-specific enhancers, we carried out motif analysis of enhancer associated sequences. *De novo* motif search led to the identification of several enriched binding motifs at each stage of BA and WA differentiation (S7 Fig). While some of the motifs are closely related to known TF binding motifs, (e.g. PPARγ in mature BA and WA) most of the motifs could not be assigned to known TFs due to our limited knowledge of the DNA binding motif for most TFs. Therefore, we examined these enhancers for the enrichment of known TF binding motifs derived from previous genome wide TF binding studies [34]. TFs with enriched motifs and robust expression at the corresponding stages are shown in Fig 3C and 3D. We found that motifs for well-known adipogenic regulators such as PPARγ, RXR, C/EBPα and FOXO1 were highly enriched in mature brown as well as white adipocytes. At the early stages, motifs for early adipogenic regulators including PBX1, KLF4 and STATs were enriched in either white or brown lineages (Fig 3C and 3D). Interestingly, the binding motif for SIX1, a homeobox transcription factor not previously implicated in the development of BAs was significantly enriched in mature BAs, but not WAs, suggesting a role for this factor in BA differentiation. To validate our finding in an *in vivo* setting, we also analyzed active enhancers in BAT and WAT tissue (using previously released datasets from ENCODE [35]) for enrichment of the SIX1 motif. Indeed, the SIX1 motif was found to be enriched at a much higher level in BAT than in WAT (S3 Table).

### Super-enhancers mark key regulators of BA differentiation

It has been shown that key cell identity genes are often associated with super-enhancers (SEs) [36], a cluster of enhancers that are enriched for binding of TFs, mediator, and chromatin marks such as H3K27ac [14]. To search for novel regulators of BA differentiation, we sought to map the SEs and define SE-associated genes in both brown and white lineages and identify common as well as lineage specific SE genes. We employed the H3K27ac ChIP-seq data to define SEs because this allowed us to monitor SEs throughout the whole process of BA as well as WA differentiation and determine the SEs present specifically at the late (d2 and d7) but not the earlier stages (d-3 to 6h). To identify genes which are potentially regulated by these SEs, we filtered for those (1) within 100 kb of the SE and (2) whose gene expression patterns correlated with SE occurrence throughout differentiation. Through this approach, we identified 419 SE-associated genes for mature BAs and 417 SE genes for mature WAs (S4 Table), of which 324 were BA selective and 322 were WA selective (Fig 3E), respectively. As expected, well-known general adipogenic marker genes such as *Cebpa*, *Fabp4 and Scd* were associated with SEs in both lineages. And SE genes at late stages of BA differentiation included most key regulators of brown adipogenesis (*Ucp1*, *Cidea*, *Fgf21* and *Ppara*) (Fig 3E and S8A Fig). Moreover, these genes tended to get transcriptionally activated (S8B Fig). Notably, *Fabp3*, *Pdk4*, *Cpt1b* and *Cpt2*, which were recently identified as putative SE associated genes in a human cell culture model of browning [10], were associated with SEs only in brown cells. Intriguingly, the TF RREB1 whose gene locus has been linked to metabolic traits like T2D susceptibility, fasting glucose levels, and body fat distribution [37–39] through genome-wide association studies (GWAS), was up-regulated during brown adipogenesis and also associated with a SE defined by H3K27ac enrichment (Fig 3F). This SE encompasses the entire promoter region of *Rreb1*. Using PPARγ binding as alternative method to define SEs in mature BAs, we found that *Rreb1* was associated with one of the top SEs in BAs due to robust PPARγ binding upstream of its transcriptional start site. In addition to *Rreb1*, other SE genes determined by PPARγ binding signals include a whole panel of key brown cell markers such as *Ucp1*, *Pgc1a*, *Cidea*, *Fgf21* and *Ppara*, and interestingly, *Pim1* (S8C Fig). Based on the above observations, we selected *Rreb1* for further functional analysis in BA development and function (detailed below).

### BMP7 activates sox genes via a p38-dependent signaling pathway during BA lineage commitment

**Fig 4:**
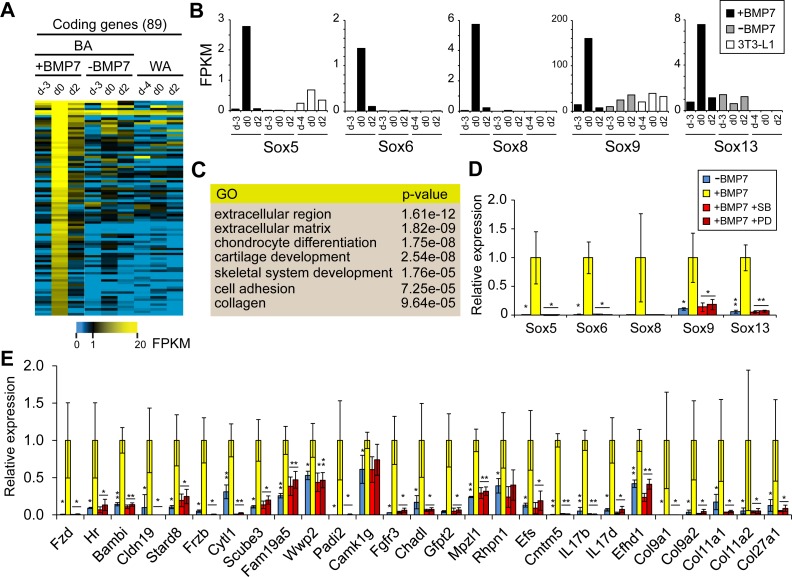
BMP7 triggers a transcriptional response during BA lineage commitment largely dependent on p38 signaling. (A) Heatmap of coding genes transiently up-regulated upon BMP7 treatment. C3H10T1/2 cells were treated with or without BMP7 for 3 days before differentiation was induced at d0 (BA). Global gene expression profiles were generated by RNA-seq at the indicated time points. 3T3-L1 cells were also included in the analysis (WA). (B) Five members of the Sox gene family were transiently induced by BMP7 treatment. (C) Gene ontology analysis of the 89 BMP7 induced coding genes from (A). (D) and (E) Blocking the p38 signaling pathway by inhibitors PD169316 (PD) or SB202190 (SB) abolished the BMP7 induced transcriptional activation of all five Sox genes (D) as well as 27 other BMP7 target genes (E). Values represent the average of three biological replicates. Error bars indicate standard deviation. *p*-values: (paired student’s t-test) * <0.05; ** <0.01.

BMP7 strongly promotes brown lineage commitment and differentiation *in vitro* and *in vivo* [24]. However, the detailed molecular mechanism underlying BMP7 function and its downstream targets during BA lineage commitment have not been thoroughly characterized. Therefore, we profiled the epigenomic and transcriptomic landscape in C3H10T1/2 cells treated with or without BMP7. To determine the molecular targets of BMP7, we compared the transcriptomic profiles between BMP7 treated and untreated C3H10T1/2 cells. On top of that, we also included the corresponding dataset from 3T3-L1 cells for comparison. To identify potential BMP7 targets, we focused on a specific group of 89 genes which showed a robust but transient induction after 3 days of BMP7 treatment (Fig 4A). Amongst those genes are modulators of WNT (*Fzd9*, *Frzb*) and TGFβ (*Bambi*, *Scube3*) signaling pathways, which were known to be involved in regulating adipogenesis (see S5 Table). In addition, the single most interesting group of genes consisted of two members of the SOX family of transcription factors, *Sox8* and *Sox13*. Using slightly relaxed cutoff criteria, we noticed that five out of the total 20 Sox genes behave as putative BMP7 targets: *Sox5*, *6*, *8*, *9* and *13* (Fig 4B). This observation suggested that at least part of the BMP7 response is mediated by the action of SOX proteins. Sox genes have been implicated in the regulation of embryonic development and in the determination of cell fate [40]. *Sox9* expression in rat MSCs increases *Cebpb* expression and favors adipogenesis [41] and *Sox5*, *6*, *and 9* play important roles in chondrogenesis. Consistent with this, we observed a transient boost of chondrogenic gene expression after BMP7 treatment and enrichment of related GO categories (Fig 4C).

BMP7 activates two major signaling pathways, the SMAD, and the p38 MAPK pathways. Previous studies suggested that p38 signaling is more important for the formation of thermogenic cells and the activation of β-adrenergic pathway [24, 42]. To determine if Sox genes are downstream targets of either the SMAD or the p38 pathway, we pre-incubated C3H10T1/2 cells with p38 inhibitors prior to treatment with BMP7, and surveyed their expression. As shown in Fig 4D, Sox gene activation was strictly dependent on p38 signaling as the treatment of p38 inhibitors PD169316 (PD) or SB202190 (SB) completely abolished the activation of all five Sox genes by BMP7. We also examined 27 additional genes from the list of BMP7 targets and found their expression was also dependent on p38 signaling (Fig 4E), which seemed to be the major transmitter of the BMP7 signal. Notably, at least two of those genes (*Col11a2 and Col9a1*) are well-known targets of *Sox9*. From the list of five Sox genes, we selected *Sox13* for further functional studies (detailed below).

In another effort to identify relevant targets from our list of 89 candidates, we defined SEs according to the H3K27ac ChIP-seq data and generated a list containing genes associated with SEs at d0 upon BMP7 treatment. When we intersected both lists we found that 14 BMP7 induced genes were indeed associated with SEs (S9A Fig) and those genes might constitute another set of important targets of the cellular response to BMP7 activation. Amongst them, we found the fibroblast growth factor receptor *Fgfr3*, which is one of the receptors for FGF21 that promotes both BAT activation and subcutaneous WAT (scWAT) browning [43]. In addition to its robust induction by BMP7, we also observed significantly elevated levels of H3K4me1, H3K9ac and H3K27ac at the upstream enhancer region and increased H3K4me3 at the promoter of *Fgfr3* gene (S9B Fig), underscoring its epigenetic regulation upon BMP7 treatment.

### PIM1, SIX1, RREB1 and SOX13 are positive regulators of BA differentiation

Using different approaches of bioinformatics analysis, we identified a number of putative regulators for brown adipogenesis. From these candidates, we selected and validated the following four factors: (i) the kinase PIM1, found to be lineage-specifically expressed in mature BAs but not WAs (Fig 2C and 2D), and three TFs, (ii) SIX1, of which the binding motif was enriched in late stage BA enhancers (Fig 3C), (iii) RREB1, associated with a SE in BAs (Fig 3F and S8C Fig), and finally (iv) SOX13, which is transiently induced by BMP7 during brown lineage commitment (Fig 4B).

**Fig 5:**
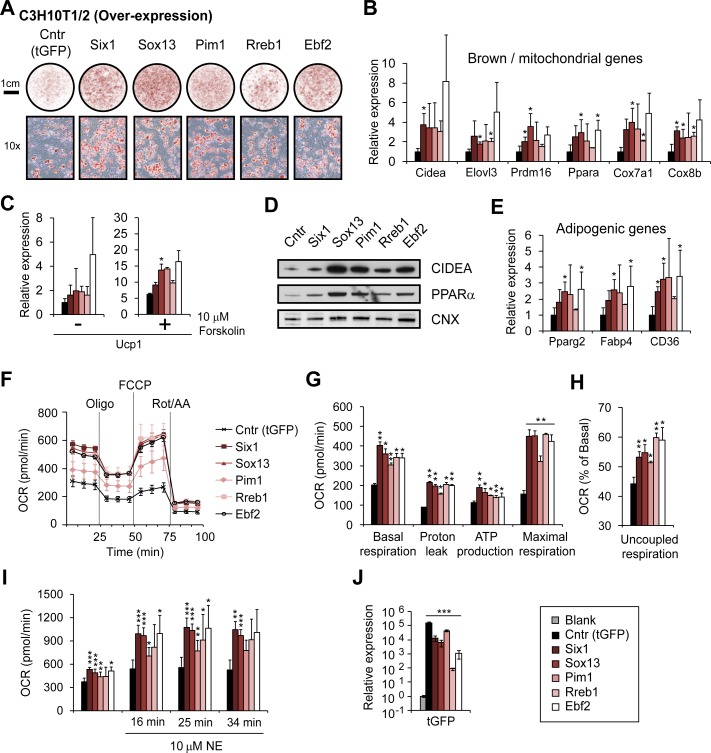
Over-expression of *Six1*, *Sox13*, *Pim1* and *Rreb1* promotes BA differentiation and function. (A) Oil-Red-O staining of C3H10T1/2 cells differentiated for 7 days without BMP7 treatment. Cells were lenti-virally transduced to over-express the indicated genes tagged with tGFP before differentiation. Cells over-expressing tGFP only or the known brown adipogenic regulator *Ebf2* served as negative and positive controls, respectively. (B) Expression of brown / mitochondrial marker genes was measured by qRT-PCR on day 7 of differentiation and found to be up-regulated upon over-expression of *Six1*, *Sox13*, *Pim1*, *Rreb1*, and *Ebf2*. (C) Expression of *Ucp1* gene in cells treated with or without forskolin. (D) Protein levels of CIDEA and PPARα were examined by Western blot at day 7 of differentiation. Calnexin (CNX) was used as a loading control. (E) Expression of general adipogenic marker genes on day 7 of adipogenesis. (F) Oxygen consumption rates (OCR) measured in mature BAs over-expressing the indicated genes. (G) Basal respiration, proton leak, ATP production and maximal respiration were determined according to the OCR values in (F). (H) Uncoupled respiration was enhanced by over-expression of *Six1*, *Sox13*, *Pim1*, *Rreb1*, and *Ebf2*. (I) Over-expression of the indicated genes enhanced the response to Norepinephrine (NE) treatment in mature BAs. (J) qRT-PCR analysis confirmed the over-expression of the transduced genes using a primer pair targeting the tGFP coding sequence. Data in panel (B), (C), (E) and (J) represent the average of three independent experiments. Panel (F)—(I) show the representative result of two independent biological replicates (assayed in quadruplets). Error bars represent standard deviation. *p*-values (paired student’s t-test): * <0.05; ** <0.01; *** <0.001

We first performed gain-of-function analysis of those factors during brown adipogenesis, by lenti-virally over-expressing the corresponding genes in the MSC line C3H10T1/2 before adipogenic induction without BMP7 treatment. As shown in Fig 5A, over-expression of each candidate, or EBF2, a known regulator of brown adipogenesis [16], significantly increased the differentiation efficiency of the C3H10T1/2 cells as monitored by Oil-Red-O (ORO) staining. Importantly, we also detected higher levels of brown / mitochondrial marker gene expression in the cells over-expressing the four candidate genes. These genes include the brown cell key regulators *Prdm16* and *Ppara*, genes involved in brown cell function (*Cidea* and *Elovl3*), and genes essential for mitochondrial activity (*Cox7a1* and *Cox8b*) (Fig 5B). Moreover, *Ucp1*, the key thermogenic gene in BAs, was also up-regulated in cells expressing the four candidate genes with or without forskolin treatment (Fig 5C). And these mRNA expression changes were reflected at the protein levels as both CIDEA and PPARα proteins were up-regulated by the over-expression of the four candidates (Fig 5D). In parallel, we also detected increased expression of general adipogenic genes such as *Pparg2*, *Fabp4* and *CD36* upon over-expression of the candidate genes, or *Ebf2* (Fig 5E). This observation was consistent with increased lipid accumulation in the corresponding cells as determined by ORO staining (Fig 5A). Increased mitochondria activity leads to up-regulated oxygen consumption rate (OCR), and this is a key feature of thermogenic brown cells. To examine the effects of *Pim1*, *Six1*, *Sox13*, and *Rreb1* over-expression on mitochondria activity, we measured the OCR in the corresponding cells 7 days after adipogenic induction. We found that both OCRs (Fig 5F) and other cellular metabolic parameters including basal respiration, proton leak, ATP production and maximal respiration (Fig 5G) were significantly increased upon candidate over-expression. In addition, we also noticed a shift towards uncoupled respiration (Fig 5H), suggesting enhanced thermogenesis. The chemical activation of the β-adrenergic pathway by drugs such as norepinephrine can significantly stimulate BAT activity. To examine the effects of candidate over-expression on the response to β-adrenergic activation, we measured the OCR in corresponding cells after norepinephrine treatment. The results showed that cells over-expressing *Pim1*, *Six1*, *Sox13*, *Rreb1*, or *Ebf2* (Fig 5J) were more susceptible to β-adrenergic activation than control cells (Fig 5I), indicating enhanced thermogenic capability. Taken together, these results suggested that all four candidates either facilitate the commitment or the differentiation process from MSCs to functional BAs.

**Fig 6:**
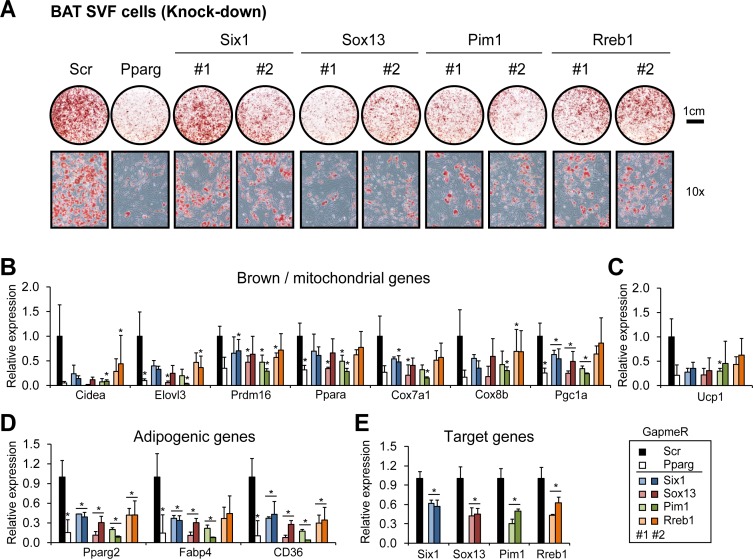
Knock-down of *Six1*, *Sox13*, *Pim1* and *Rreb1* impairs brown adipogenesis. (A) Oil-Red-O staining of SVF cells isolated from BAT after *in vitro* differentiation for 7 days. Before adipogenic induction, cells were transfected with two independent (#1 and #2) locked nucleic acid (LNA) longRNA GapmeRs targeting *Six1*, *Sox13*, *Pim1*, or *Rreb1*. Scrambled (Scr) or *Pparg*-targeting LNA GapmeRs served as negative and positive controls, respectively. (B) Expression of brown / mitochondrial marker genes was measured by qRT-PCR on day 7 of differentiation and found to be down-regulated upon knock-down of *Six1*, *Sox13*, *Pim1*, *Rreb1* and *Pparg*. (C) Expression of *Ucp1* was down-regulated upon knock-down of the indicated genes. (D) Expression of general adipogenic genes on day 7 of differentiation. (E) mRNA levels of the targeted genes were assayed 24-hour post transfection. Panel (B)-(E) summarize the average of three independent experiments. Error bars represent standard deviation. *p*-values (paired student’s t-test): * <0.05.

We showed that *Pim1*, *Six1*, *Sox13* and *Rreb1* were sufficient to promote brown adipogenesis. To test whether they were also necessary for BA differentiation, we performed loss-of-function analysis using Stromal Vascular Fraction (SVF) cells isolated from BAT transfected with LNA longRNA GapmeR oligonucleotides to knock down the genes of interest before adipogenic induction. As shown in Fig 6A, cells transfected with a scramble oligo (Scr) readily differentiated into mature BAs, whereas knock-down of either the candidate genes using two independent GapmeRs or *Pparg* led to severely reduced capabilities to differentiate as demonstrated by ORO staining. In addition, prominent brown */* mitochondrial regulators and markers such as *Prdm16*, *Pgc1a*, *Ppara*, *Cidea*, *Elovl3*, *Cox7a1* and *Cox8b* (Fig 6B), as well as *Ucp1* (Fig 6C) were down-regulated in cells with *Pim1*, *Six1*, *Sox13*, and *Rreb1* knock-down. Consistent with the ORO staining results, adipogenic markers including *Pparg2*, *Fabp4* and *CD36* were also reduced (Fig 6D) by the knock-down of the candidate genes (Fig 6E). We also validated the function of the four candidates in SVF cells isolated from posterior scWAT. Those cells have a certain capacity to “brown” [44] and over-expression or knock-down of the four candidates resulted in similar outcomes as observed in the MSC and BAT SVF cell systems (S10 and S11 Figs).

### SIX1 binds to brown marker genes and interacts with key regulators of brown adipogenesis

**Fig 7:**
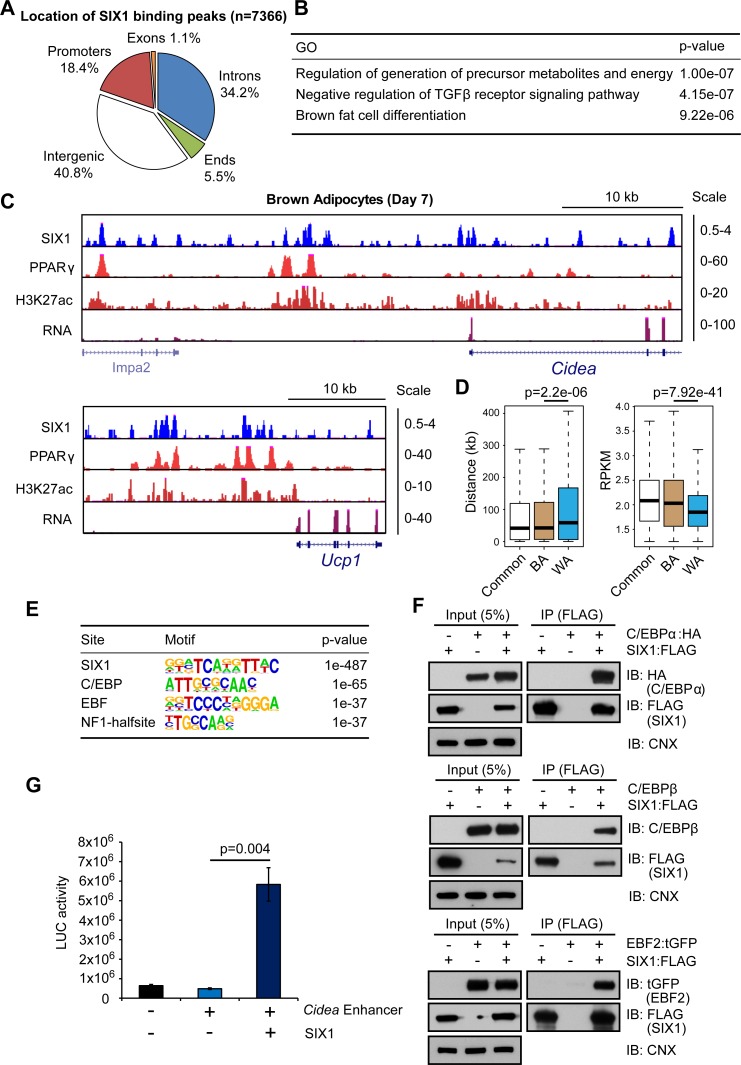
Genome-wide SIX1 binding analysis in mature BAs. (A) ChIP-seq analysis of SIX1 binding revealed that it binds preferentially to intergenic regions, promoters and introns. (B) Gene ontology analysis of genes associated with SIX1 binding peaks. (C) SIX1 binds to the *cis*-regulatory regions of the brown marker genes *Cidea* and *Ucp1*. PPARγbinding, H3K27ac and mRNA expression profiles are also shown at these loci. (D) Box plots comparing SIX1 binding signals (RPKM) at BA-specific, WA-specific and commonly expressed genes, and distances of the closest SIX1 binding site to the transcription start site of BA-specific, WA-specific and commonly expressed genes. (E) Motif analysis of SIX1 binding sites revealed enrichment of C/EBP, EBF and NF1 binding motifs. (F) Co-immunoprecipitation experiments confirmed physical interactions between SIX1 and C/EBPα, C/EBPβ, as well as EBF2 in HEK293 cells. (G) Luciferase assay in HEK293 cells transfected with a plasmid carrying the luciferase gene driven by a portion of the *Cidea* distal enhancer (see S12 Fig for details) with or without SIX1 over-expression. n = 3, Error bars represent standard deviation. *p*-values were calculated using the paired student’s t-test.

To gain further mechanistic insight into the mode of action for one of the identified factors, SIX1, we mapped its genomic localization via ChIP-seq in mature BAs. We found in total 7366 binding peaks for SIX1 with most of them located at intergenic regions, introns and promoters (Fig 7A), which is typical for TFs [45]. GO analysis of the SIX1 binding genes revealed that “regulation of generation of precursor metabolites and energy”, “negative regulation of TGFβ receptor signaling pathway” and “brown fat cell differentiation” were amongst the most significantly enriched categories (Fig 7B). We detected SIX1 binding at the *cis*-regulatory regions (marked by H3K27ac) of brown markers such as *Cidea* and *Ucp1* (Fig 7C). Moreover, we observed partial overlap of SIX1 binding to PPARγ binding at these regions. In a more quantitative analysis we measured the strength and proximity of SIX1 binding at brown-specific, white-specific and commonly expressed (i.e. white and brown) genes. We found that SIX1 bound preferentially around brown-specific and commonly expressed genes as compared to white-specific genes, suggesting a role for this factor in regulating brown selective as well as general adipogenic gene expression (Fig 7D). To decipher the molecular mechanism underlying SIX1 function, we performed a motif analysis of SIX1 bound regions. As expected, the most enriched binding motif was for SIX1 itself, which was followed by motifs for C/EBP, EBF, and NF1 TFs (Fig 7E). PPARγ and RXR binding motifs were only mildly enriched. The enrichment of C/EBP and EBF binding motifs at SIX1 binding sites suggested physical interactions between these TFs. Indeed, we verified the direct interactions between SIX1 and C/EBPα, C/EBPβ, as well as EBF2 using co-IP assays (Fig 7F). Finally, using luciferase activity assay, we found that an upstream enhancer element of the *Cidea* gene harboring a SIX1 motif (S12 Fig) promotes expression in a SIX1-dependant manner (Fig 7G). Together, our findings corroborate a model in which SIX1 can be recruited to brown-specific or general adipogenic genes through either direct DNA binding (via the SIX1-binding motif) or recruitment by EBF2 and C/EBP proteins (at regions with no SIX1-binding motif).

## Discussion

Promoting energy expenditure through thermogenesis is of significant interest as potential therapy for obesity and related diseases. It requires the recruitment of thermogenic fat cells such as brown and beige/brite adipocytes. Existing evidence suggests that the majority of these thermogenic cells are recruited *de novo* in response to environment cues [43, 46–48]. Therefore, to promote thermogenic adipocyte recruitment, it is essential to have a fundamental understanding of the gene regulation network that governs brown adipogenesis, especially at the lineage commitment step. In this study, we provide comprehensive profiles of the transcriptome and epigenome at five key developmental stages throughout the differentiation of murine multi-potent MSCs into mature BAs. Through in-depth bioinformatics analyses, we identified and functionally validated PIM1, SIX1, RREB1, and SOX13 as novel regulators promoting brown cell differentiation and function.

Differential gene expression analysis is a classic approach for the identification of regulators of cell type specification. A number of adipogenic and brown fat cell regulators including PPARγ, C/EBPα and PRDM16 were identified through this approach. In our study, we also used this analysis to identify brown selective genes but added additional criteria for the selection of candidates: these genes must be dynamically regulated during adipogenesis and stage-specifically expressed only in mature adipocytes (Fig 2). As the result, our list of 121 brown selective genes contains brown markers such as *Cidea* (#1), *Elovl3* (#3), *Ucp1* (#24) and *Ppara* (#61), as well as a number of mitochondrial genes including *Cpt1b* (#4). From this list, we specifically looked for factors that could potentially be involved in gene regulation or signal transduction, and we selected the kinase PIM1 (#53) for further analysis. Moreover, the *Pim1* gene was later found to be associated with a SE in brown cells (S8C Fig). In our study, over-expression of *Pim1* in both C3H10T1/2 cells and the scWAT SVF cells up-regulated a number of key brown cell marker genes as well as general adipogenic genes (Fig 5B–5E and S10B–S10D Fig). In addition, over-expression of this kinase also promoted the mitochondrial respiration in general and specifically uncoupled respiration, a feature of thermogenic fat cells (Fig 5F–5H and S10F and S10G Fig). In contrast, knock-down of *Pim1* by GapmeRs reduced the expression of brown marker genes and adipogenesis efficiency in both primary brown cells (Fig 6) and subcutaneous white cells (S11 Fig). Therefore our analysis clearly implicates *Pim1* in brown adipogenic differentiation, although future experiments will have to address if its role is solely restricted to the brown lineage. With our experimental model we cannot rule out that enhanced brown differentiation is partially caused by an increase in adipogenic differentiation in general. However, the lineage specific expression of *Pim1* in BAT *vs* WAT, together with its increased expression in BAT upon cold exposure (Fig 2D), point towards a more specific role in BAT for this kinase. It will be interesting to investigate the functional significance and molecular mechanism of PIM1 in different tissues and under different metabolically challenging conditions such as diet induced obesity or cold exposure *in vivo*, especially the direct targets of this kinase.

Enhancer binding motif analysis is another powerful tool to identify novel TFs involved in specific cell differentiation processes [15, 16]. In our study, we first defined stage-specific enhancers during both white and brown adipogenesis, then surveyed the enrichment of TF binding motifs at early and late stages of differentiation. While enrichment for several well-known adipogenic factors at late stages of both brown and white adipogenesis was expected, our finding that the motif for the TF SIX1 was enriched during late brown adipogenesis was surprising (Fig 3C), since SIX1 has not been implicated in brown cell differentiation so far. *Six1* belongs to the *Si**ne Oculis Homeobo**x* family of genes and has been reported to play a crucial role in muscle cell lineage decision as well as muscle development. Through gain- and loss-of-function assays, we confirmed that *Six1* was required for the expression of brown selective and adipogenic marker genes in both the C3H10T1/2 cells and the SVF cells from scWAT and BAT. Moreover, over-expression of *Six1* enhanced the mitochondrial uncoupled respiration in differentiated C3H10T1/2 cells (Fig 5H). In an attempt to decipher SIX1’s mode of action, we performed genome-wide binding profiling of SIX1 in mature BAs. Strikingly, we found that SIX1 bound to brown as well as general adipogenic genes, some of whose expression were affected by the modulation of the *Six1* gene, suggesting that these genes are direct targets of SIX1. Through binding motif analysis of SIX1 occupied regions and subsequent co-IP assay, we confirmed that SIX1 directly interacts and may cooperate with C/EBPα, C/EBPβ and EBF2 in regulating the transcription network during differentiation. Therefore the role of SIX1 seems not to be restricted to the regulation of BA differentiation, but it may act as a more general activator of the adipogenic transcriptional program. Again, *in vivo* studies to investigate the physiological role of *Six1* in different adipogenic tissues will be instrumental to fully understand its biological function.

Super-enhancers are big clusters of enhancers and are often associated with genes specifying cell identity [14]. It can be defined by either mediator or TF binding or the enrichment of chromatin marks such as H3K27ac. Through analysis of super-enhancer associated genes, KLF11 was identified as an important factor promoting the browning of human mature white fat cells [10]. In our study, we profiled the stage-specific super-enhancers in a distinct process: i.e. the differentiation of brown adipocytes from multi-potent progenitor cells. That way we identified 419 genes associated with mature BA specific super-enhancers, including most brown marker genes. From this gene list, we selected the TF RREB1 for further functional analysis on the basis of its link to metabolic traits and association with one of the highest ranking SEs (#8) in BAs as defined by PPARγ binding. In our functional studies, over-expression of *Rreb1* led to increased expression of brown marker genes and enhanced mitochondrial respiration in both C3H10T1/2 cells and SVF cells from scWAT. On the other hand, knock-down of *Rreb1* resulted in reduced brown marker expression and impaired adipogenesis of SVF cells from BAT and scWAT. Moreover, RREB1 was very recently identified as a positive regulator of brown adipogenesis via a distinct bioinformatics approach [32] during the preparation of this manuscript. Further physiological studies on the role of *Rreb1* in insulin sensitivity and energy homeostasis using corresponding gain- and loss-of-function animal models will be necessary to fully characterize the function of this positive regulator of brown adipogenesis.

BMP7 was recently established as a key factor that specifies the brown lineage from MSCs [24]. Mechanistically, BMP7 acts through either the SMAD or the p38 MAPK signaling pathway to induce its downstream target genes governing the adipogenic or thermogenic program. In order to gain an in-depth understanding of the brown lineage commitment from multi-potent progenitor cells (as exemplified by BMP7 signaling), we systematically compared the gene expression profiles of C3H10T1/2 cells with or without BMP7 treatment. We found that 89 genes were transiently induced by BMP7 treatment, amongst which we identified a panel of Sox genes. We found that those genes were all p38/MAPK dependent, suggesting their involvement in promoting the thermogenic rather than the general adipogenic program. Subsequent functional analysis confirmed that one candidate gene, *Sox13*, promotes adipogenic differentiation, brown marker gene expression, and mitochondrial respiration. Our current results suggest that BMP7 triggers an early p38 dependent response, including the activation of Sox gene expression, important for the lineage commitment of brown cells from multi-potent progenitor cells. Clearly, future work is needed to dissect the exact contribution of the different Sox genes during this process and to define their downstream target genes.

Transcriptomic profiles and epigenomic landscapes are important resources for understanding the gene regulation network in a certain cell type or in a specific cell differentiation process. Analysis of those datasets has led to the identification of numerous novel regulators in various cellular processes. Seminal works in the field have provided valuable resources for further investigation of the molecular control of fat cell differentiation [10, 15, 23, 49–53]. Here we add a comprehensive study of the epigenomic and transcriptomic transitions at five key developmental stages throughout the process of murine brown adipogenesis. Our dataset comprises a high temporal resolution of the differentiation process as well as a broad coverage of chromatin marks. Through comparative analyses of white and brown datasets using various bioinformatics tools, we identified many potential candidates and validated four factors that promote thermogenic adipocyte differentiation in various cellular models including C3H10T1/2 cells, SVF cells from subcutaneous WAT and interscapular BAT. Moreover, through analyzing the chromatin dynamics at the promoters of lineage-specific and commonly expressed adipogenic genes in BA and WA, we found that, in addition to the mechanism proposed by a recent study [32], which suggested that the removal of H3K27me3 is required for brown gene expression, the pre-deposition of H3K4me1 at these genes during early stages of brown adipogenesis is essential for poising them for expression at a later stage. For general adipogenic genes, we found they are only marked by H3K4me1 but not H3K27me3 during both brown and white adipogenesis, suggesting their activation does not involve H3K27 demethylation. Based on these observations, we propose that the pre-deposition of H3K4me1 at brown specific genes is a critical step in the chromatin remodeling during the process of brown adipocyte lineage commitment, while the full activation of these genes is only possible once the “stop sign” (H3K27me3) is removed. Besides proposing this conceptual model for the epigenetic regulation of brown lineage specific genes, we anticipate additional factors (including lncRNAs and miRNAs), promoting or inhibiting brown cell differentiation to be identified through analyzing these datasets and further insights to be gained from these resources.

## Materials and Methods

### Cell culture

3T3-L1 preadipocytes and C3H10T1/2 mesenchymal stem cells were purchased from ATCC. 3T3-L1 cells were maintained in DMEM (Gibco, 11995–065) supplemented with 10% bovine calf serum (BCS; HyClone, SH30072.03). For 3T3-L1 differentiation, BCS was replaced by 10% fetal bovine serum (FBS; HyClone, SH30070.03) and cells were seeded on gelatinized dishes to reach 70% confluency on the next day (d-4). Two days later cells reached full confluency (d-2) and after another two days (d0) differentiation was induced by adding 1 μM dexamethasone (Sigma, D4902), 0.5 mM 3-isobutyl-1-methylxanthine (Sigma, I7018), and 10 μg/ml insulin (Santa Cruz, sc-360248) for two days. Subsequently cells were maintained in DMEM with 10% FBS and 10 μg/ml insulin; medium was changed every other day.

To differentiate C3H10T1/2 cells into brown adipocytes, cells were maintained in DMEM (high glucose) supplemented with 10% Fetal Clone III serum (Hyclone, SH 30109.03), split onto gelatinized dishes at 70% confluency the next day (d-3) and treated with 8.3 nM human recombinant BMP7 (R&D Systems, 354-BP) for three days. At day 0, differentiation was induced by adding 5 μM dexamethasone, 0.5 mM 3-isobutyl-1-methylxanthine, and 0.12 μg/ml insulin, 1 μM rosiglitazone (Cayman, 71740) and 1 nM 3,3’,5-Triiodo-L-thyronine (Sigma, T5516) to the medium. From day two on the cells were maintained in DMEM, 10% FetalClone III with 0.12 μg/ml insulin, 1 μM rosiglitazone and 1 nM 3,3’,5-Triiodo-L-thyronine. The medium was replaced every two days.

To test the effect of p38 inhibition, 10 μM PD169316 (GenEthics) or 10 μM SB202190 (Sigma) was added to the medium six hours prior to BMP7 treatment for three days.

SVF cells were isolated from 8 weeks old male C57BL/6J mice. Brown adipose tissue from the interscapular region or posterior subcutaneous white adipose tissue was collected, rinsed in 1x HBSS (Gibco, 14175) supplemented with 50 μg/ml D-Glucose (Sigma, G8644), cut into small pieces, digested with 3 ml collagenase solution (Collagenase 1 mg/ml, Sigma, C9891; BSA 20 mg/ml, Sigma-Aldrich, A7906; D-Glucose 50 μg/ml, Sigma, G8644; in 1xHBSS) per 1 g of tissue for 1–1.5 hours by nutating at 37°C. The digestion was stopped by adding SVF growth medium (DMEM/F12, Gibco, 11330–032; 20% FBS, Hyclone; PenStrep, Gibco, 15140–122). Cells were pipetted up and down carefully, centrifuged for 5 min at RT at 400*g*. After careful resuspension of cells in fresh growth medium, they were passed through a 100 μM filter mesh (BD Falcon, 352360), centrifuged again, and resuspended in 1xHBSS supplemented with 50 μg/ml D-Glucose. The washing step was repeated twice. After the last wash, cells were resuspended in 1x Red Blood Lysis buffer (Biolegend, 420301), incubated for 5 min at RT before another centrifugation. Finally, cells were resuspended in growth medium and plated onto cell culture plates. Cells were expanded in growth medium and differentiation started not later than passage four. For adipogenic differentiation, cells were seeded on gelatinized 12-well plates (40,000 cells/well) in differentiation medium (DMEM/F12; 10% FBS). Two days later the medium was replaced by induction medium (DMEM/F12; 10% FBS; 5 μM dexamethasone, 0.5 mM 3-isobutyl-1-methylxanthine, 0.5 μg/ml insulin, 1 μM rosiglitazone, and 1 nM 3,3’,5-Triiodo-L-thyronine). After three days the medium was changed to differentiation medium supplemented with 0.5 μg/ml insulin and 1 nM 3,3’,5-Triiodo-L-thyronine and replaced every other day.

### Lenti-viral over-expression

Plasmids containing full length cDNA were purchased from OriGene (hSix1 (RC203465), hSox13 (RC210697), hPim1 (RC205853), mEbf2 (MR224591)) or Addgene (hRreb1 (41145)), and sub-cloned into a lenti-viral vector providing an EF1a promoter for expression and a C-terminal tGFP-tag (OriGene PS100072). In order to not exceed the packaging capacity of the lenti-virus, the C-terminal portion of hRreb1 (the last 333 amino acids) was removed, leaving behind a truncated version of Rreb1, hRreb1ΔC (1–1408 amino acids). Lenti-viral particles were produced in HEK293NT cells using the Lenti-vpack lenti-viral packaging kit from OriGene (TR30022) following the manufacturer’s protocol. C3H10T1/2 cells or SVF cells were transduced with lenti-viral supernatant diluted 1:1 in fresh growth medium and 8 μg/ml Polybrene overnight.

### Transient knock-down

The following LNA-longRNA GapmeRs (300600) from Exiqon were used to knock down the indicated genes: mSix1 #1 (CAAACTGGAGGTGAGT), mSix1 #2 (CAGAGGAGAGAGTTGA); mSox13 #1 (GCAAAGGCTGGTGGCT), mSox13 #2 (GAGGAGGAGGTTTAGC); mPim1 #1 (GGAGTTGATCTTGGAC), mPim1 #2 (GGTGATAAAGTCGA); mRreb1 #1 (GTTAGATTTGGTAGA), mRreb1 #2 (CGTTGATGAGAGGTG); Pparg (AGAAATCAACTGTGGT); scr (/56-FAM/AACACGTCTATACGC). Prior to transfection, the SVF cells were seeded on gelatinized 12 well plates (40,000 cells/well). On the next day, cells were transfected with 60 μM LNA-longRNA GapmerRs using 4.5 μl Lipofectamine 2000 (Invitrogen, 11668019).

### Cellular flux assay

To determine cellular oxygen consumption rates, we used the Seahorse XFe24 Extracellular Flux Analyzer. Cells were seeded on gelatinized XFe24 cell culture microplates (100777–004) at 4,000 cells/well and differentiated one day post confluency following the procedures described above. The XF Cell Mito Stress Test Kit (103015–100) was used to determine basal respiration, ATP production, proton leak, maximal respiration, and spare respiratory capacity. Concentrations of the added chemicals were: 40 μM oligomycin, 0.15 μM FCCP, 1 μM rotenone / 1 μM antimycin A. To activate the β-adrenergic pathway before measurement, cells were treated with 10 μM norepinephrine (Sigma Aldrich, A0937).

### Gene expression analysis

Cells were harvested with TRIzol reagent (Ambion, 15596018) and total RNA was extracted following the manufacturer’s protocol. RNA was treated with Amplification Grade DNase I (Invitrogen, 18068–015) before the generation of either RNA-seq libraries or cDNA to assess expression of individual genes. RNA-seq libraries were generated by BGI, China, following standard procedures. For cDNA generation, 500 ng of total RNA were reverse transcribed using random 9-mers and M-MLV Reverse Transcriptase (Invitrogen, 28025–021). Target gene expression was determined via quantitative Real-Time PCR analysis using Power SYBR Green PCR Master Mix from Applied Biosystems (4367659) on a 7900HT Fast Real-Time PCR machine (Applied Biosystems). All gene expression data in this study was normalized to the expression of the riboprotein gene 36B4 unless indicated differently. Primers were listed in S6 Table.

### ChIP-seq

Chromatin immunoprecipitation was performed as described earlier [54]; antibodies used in this study were listed in S6 Table. To construct ChIP-seq libraries, we employed a method described previously [55]. In short, 5ng of ChIP DNA were used as starting material. After an end repair step, terminal addition of poly-dCs, and ligation of linkers, the DNA was amplified in a two-step PCR procedure. The final product was loaded onto a 2% agarose gel and fragments between 200bp and 500 bp were cut from the gel, purified and sequenced at BGI China.

### Western blotting

Cells were lysed using a modified version of the RIPA buffer (50 mM Tris-HCl pH8, 0.5% NaDeoxycholate, 150 mM NaCl, 1% NP-40, 0.1% SDS) supplemented with proteinase inhibitor cocktail mix (Roche, 11 873 580 001), sonicated (Bioruptor, 10 cycles, 10 seconds, high energy), and centrifuged for 15 minutes at 4°C. The cell lysates (supernatants) were quantified using Bradford reagent (Bio-rad, 500–0205), separated on the Bio-rad Minigel system, and blotted onto nitrocellulose membranes (Bio-rad, 162–0115), which were blocked using StartingBlock T20 blocking buffer from Thermo Scientific (37543). Antibodies used were listed in S6 Table. Signals were detected using the WesternBright ECL detection kit from Advansta (K-12045-D20).

### Co-immunoprecipitation

HEK293 cells were transfected using Lipofectamine 2000 (Invitrogen) with the following plasmids: pCMV6-hSix1:myc:DDK from Origene (RC203465); pcDNA3.1-Cebpa:HA, a kind gift from Dr. D. Tenen; pcDNA3.1-mCebpb from Addgene (12557); pLenti-EF1ap-mEbf2:tGFP (Origene). Three days after transfection, cells were harvested and lysates were prepared as for Western blots except that the lysis buffer contained 50 mM Tris-HCl pH7.4, 150 mM NaCl, 1 mM EDTA, 0.1% NP40, and proteinase inhibitor mix (Roche, 11 873 580 001). 500 μg of total proteins were pre-cleared with 10 μl protein G beads (Sigma, P3296) and incubated overnight with 10 μl anti-FLAG M2 affinity gel at 4°C on a rotator (Sigma, A2220). The gel was washed twice each with lysis buffer, RIPA buffer containing 0.1% SDS, RIPA buffer containing 0.1% SDS and 1% NP-40. Precipitated proteins were eluted using SDS loading buffer (95°C, 5 min) and separated on PAGE gels for Western analysis.

### Luciferase assay

A 222 bp fragment corresponding to the region -13,982 to -13,761 from the *Cidea* transcription start site (TSS) was PCR-amplified from mouse genomic DNA and cloned into pGL3-Basic plasmid (Promega) to yield pGL3-Cidea-Enh1. For the luciferase assay, HEK293 cells were transfected with pGL3-Cidea-Enh1 (or equal amounts of pGL3-Basic) and pCMV6-hSix1:myc:DDK from OriGene (RC203465) (or equal amounts of pCMV6:myc:DDK) using Lipofectamine 2000 (Invitrogen). 48h after transfection cells were lysed and Firefly luciferase activity was measured on a GloMax Multi luciferase reader using the Dual-Glo luciferase assay kit (Promega) following the manufacturer’s recommendations.

### Oil-Red-O staining

Cells were washed with 1x PBS, fixed with 10% formaldehyde (Sigma-Aldrich, 252549) for 20 minutes at room temperature, washed three times with 1x PBS, and incubated with freshly made Oil-Red-O working solution (60% Oil-Red-O stock solution [5 mg/ml Oil-Red-O (Sigma-Aldrich, O0625) in 60% triethylphosphate solution] and 40% dH2O) for 1h. Finally, cells were rinsed with dH2O.

### ChIP-seq data analysis

All ChIP-seq datasets were aligned using Bowtie (version 2.0.4) to build version mm9 of the murine genome. Alignments were performed with the following additional parameters: -t -q -p 8 -N 1 -L 25. To visualize the ChIP-seq signals for each histone modification and PPARγ, we extended each read to 300 bp and counted the coverage of each read for each base, which was shown as the UCSC genome browser tracks. For the downstream analysis, we normalized the read counts for the ChIP samples by computing the numbers of Reads Per Kilobase of bin per Million reads sequenced (RPKM). To minimize the batch and cell type variation, the RPKM values were further normalized through Z-score transformation. MACS [56] was used to identify histone modification regions and PPARγ binding peaks by default settings.

### RNA-seq data analysis

All RNA-seq datasets were aligned using Tophat (version 2.0.6) to build version mm9 of the murine genome. Alignments were performed with the following additional parameters:-p 2—solexa1.3-quals. The mapped reads were further analyzed by Cufflinks (v 2.1.1) [57] and the expression levels for each transcript were quantified as Fragments Per Kilobase of transcript per Million mapped reads (FPKM) based on refFlat database.

### Identification of differentiation stage-specific coding genes, lncRNAs and microRNAs

To identify differentiation stage-specific coding genes, we used a strategy described previously based on the Shannon entropy to compute a stage-specificity index to all expressed coding genes [58–60] using the averages of two RNA-seq replicates. The entropy score for each gene was defined as described as follows [61]: for each gene, we defined its relative expression in a cell type i as Ri = Ei /∑E, where Ei is the FPKM value for gene expression in the cell type i; ∑E is the sum of FPKM values in all cell types; N is the total number of cell types. Then the entropy score for this gene across cell types can be defined as H = -1*∑Ri * logRi (1≤i≤N), where the value of H ranges between 0 to log2(N). An entropy score close to zero indicates the expression of this gene is highly stage-specific, while an entropy score close to log2(N) indicates that this gene is expressed ubiquitously. Based on an examination of the entropy distribution genes with entropy less than 2 were selected as the candidate stage specific genes. Among these candidates, we selected genes for each stage based on the following criteria: the gene was highly expressed in this stage (FPKM>5), and high expression (FPKM>5) could not be observed in more than three additional stages. These genes were then reported in the final stage specific coding gene list and used for subsequent analyses, e.g. GO analysis (see below).

To identify stage specific lncRNAs, we used NONCODE v4 mouse annotation database [26] and removed the regions overlapping with refFlat annotation. The expressed putative lncRNAs are those with FPKM>0.5 in at least one of the five differentiation time points for each lineage. We used entropy score less than 2 to select candidates of stage specific lncRNA and applied the following criteria: the lncRNA was highly expressed in this stage (FPKM>0.5), and expression (FPKM>0.5) could not be observed in more than three additional stages for this lncRNA. These lncRNAs were then reported in the final stage specific lncRNA list.

The microRNA array signals were quantile normalized among different stages during adipogenesis and we selected those with expression varying by more than two-fold between different stages during adipogenesis. They were assigned to their specific stages based on their maximum expression.

### Identification of day 7 adipocyte specific coding genes and lncRNAs

Day 7 adipocyte specific coding genes were identified from the day 7 stage specific gene list obtained above. We selected the brown d7 adipocyte specific genes based on the following criteria: (1) brown adipocyte d7 expression was at least 3 fold higher than that in white d7 adipocytes; (2) this genes was not highly expressed in white d7 adipocyte (FPKM<10). To generate the list of lineage-specific genes in Fig 2C, we introduced two additional criteria: (1) expression in differentiated SVFs from BAT was at least 2.5-fold higher than in differentiated SVFs from WAT (data from [27]), and (2) expression in BAT tissue was at least 2.5-fold higher than in WAT tissue (harvested from male C57BL6 mice). The criteria for white specific genes were *vice versa*. Day 7 adipocyte specific lncRNAs were identified from the day 7 stage specific lncRNA list obtained above. To be defined as brown specific lncRNAs, their expression (FPKM) at day 7 had to be at least 5-fold higher in brown than that in white adipocytes. The criteria for white d7 adipocyte specific lncRNAs were *vice versa*.

### Identification of stage specific enhancers

To identify enhancers (without overlapping promoters) we first called H3K27ac and H3K4me3 peaks for each stage by MACS. The peaks from all stages (BA: d-3, d0, 6h, d2, d7; WA: d-2, d0, d2, d7) were pooled for individual modifications and lineages. Enhancers were identified from the H3K27ac regions not overlapping with H3K4me3 regions and regions 2.5 kb up- and down-stream of Refseq transcription start sites. We calculated the RPKM within these enhancer regions and their normalized signals by Z-score normalization. Stage specific enhancers were defined based on the following criteria: the enhancer was highly active in this stage (normalized RPKM>1), and its high activity (normalized RPKM>0) could not be observed in more than three additional stages. These enhancers were then reported in the final stage specific enhancer list.

### Identification of lineage specific coding genes and determination of the chromatin state at their promoters

We first extracted BA d7 and WA d7 stage specific genes as described above. Those genes were first pooled, and then separated into BA-specific, WA-specific and shared common adipogenic genes using the following criteria: a gene was defined as BA-specific if (1) brown adipocyte d7 expression was at least 3 fold higher than that in white d7 adipocytes; (2) this gene was not highly expressed in white d7 adipocyte (FPKM<10). The criteria for WA-specific genes were vice versa. The rest of the genes were identified as shared genes. Then we calculated the z-score normalized RPKM of each histone modification on promoter regions. Then all these normalized RPKM signals were mapped to its corresponding genes. The box plots show the normalized RPKM of different histone modification on different groups of genes. p-values were calculated using t-test.

### Identification of super-enhancers and their potential targets

Super-enhancers of brown adipocyte and white adipocyte were identified by the software ROSE [36] based on our brown adipocyte d7 PPARγ ChIP-seq data and published 3T3-L1 d7 PPARγ ChIP-seq data. The closest gene to the super-enhancer was assigned as its potential target. Alternatively, SEs were identified using the H3K27ac datasets. To do this, we calculated Z-score normalized RPKM values on enhancer regions identified above and selected active enhancers with normalized RPKM >0 within 12.5 kb to further merge nearby signals into ‘stitched’ enhancers. We kept the ‘stitched’ enhancers larger than 12.5 kb as super-enhancer candidates. The RPKM values were calculated on these new super-enhancer candidates and ranked to find the super enhancers with slope > 0.5. To identify SEs specific to late BAs or late WAs, we used the corresponding d7 SEs and removed SEs which showed an overlap with SEs also present at early stages (i.e. d-3, d0, 6h for BA and d-2, d0 for WA). We assigned super enhancers to expressed genes (FPKM>1) within 100 kb of super-enhancer based on the Pearson correlation between expression and enhancer activity during adipogenesis. Genes with correlation coefficient higher than 0.75 were selected as the potential super-enhancer targets. If there was no gene with more than 0.75 correlation coefficiency within 100 kb of a super-enhancer, we assigned the gene with the largest correlation coefficiency above 0.5 as its potential target. microRNAs within 100 kb of super-enhancer were all assigned as potential targets.

### Motif analysis

To find the sequence motif enriched in the identified enhancers, we used the Homer [34] program based on mm9 genome using default settings. For motif analysis, background sequences were randomly selected from the genome, matched for GC% content to facilitate subsequent GC normalization.

### Gene ontology analysis

To find enriched GO categories, we used the DAVID web-tool (using default settings) [62] with Gene Ontology database including Molecular Function terms, Biological Function terms and Cell Component terms. To find the GO categories enriched around enhancers, super-enhancers and TF binding peaks, we used the GREAT tool [63] using default settings. Background calculation was based on the whole genome.

### Distribution and enrichment of six1 binding peaks

Six1 peaks were called by MACS as described above. The overlap between the peaks and annotated genome elements was calculated based on the mm9 genome annotation. To calculate Six1 enrichment, WA-specific, BA-specific and common genes were defined as follows: WA-specific genes: FPKM>5 in WA d7 and FPKM<5 in BA d7; BA-specific genes: FPKM>5 in BA d7 and FPKM<5 in WA d7; common genes show FPKM>5 in both WA d7 and BA d7. When comparing the Six1 signal around those groups of genes, we first defined an unbiased set of regulatory sites and potential Six1 binding site around the TSS using H3K27ac peaks from brown adipocytes (d7), H3K27ac peaks from white adipocytes (d7) or shared H3K27ac peaks from both brown and white adipocytes. Overlapping Six1 peaks were counted and enrichment was calculated as RPKM by adding the signals of Six1 peaks. Distances between genes and their closest Six1 binding sites were based on their annotated TSSs.

### Accession numbers

All ChIP-seq, RNA-seq and microRNA microarray datasets were deposited in GEO under the accession number of GSE75698.

### Ethics statement

All animal procedures were performed according to a protocol (IACUC#130829) approved by the Institutional Animal Care and Use Committee of the Agency for Science, Technology and Research (A*STAR) of Singapore.

## Supporting Information

S1 Fig Validation of the *in vitro* tissue culture models of BA and WA differentiation. **Related to Fig 1.** (A) Oil-Red-O staining of the cells at various differentiation stages during C3H10T1/2 (BA) and 3T3-L1 (WA) adipogenesis. (B) Morphology of C3H10T1/2 and 3T3-L1 cells at key stages of BA and WA differentiation. (C)-(D) Expression of (C) general adipogenic and (D) brown / mitochondrial marker genes before (d0) and after (d7/d8) differentiation. Data were normalized to Peptidylprolyl Isomerase A (*Ppia*) expression; graphs summarize the results of 3 technical replicates, error bars represent standard deviations. (E) Western blot confirmed the expression of PPARγ protein in mature BA and WA cells, whereas UCP1, PPARα, and CIDEA proteins were only detected in mature BAs. Hexokinase was used as a control for mitochondrial content. Calnexin served as a loading control. The asterisk (*) marks an unspecific band in the UCP1 blot.
(TIF)

S2 Fig Reproducibility of the histone modification ChIP-seq datasets and chromatin dynamics at gene promoters during brown adipogenesis. **Related to Fig 1.** (A)-(B) Heatmaps of H3K4me1, H3K4me3, H3K9ac, H3K27ac and H3K27me3 ChIP-seq signals at the promoter region of annotated genes at (A) d0 and (B) d7 in BAs. Note that the two independent replicates (rep1 and rep2) are highly consistent. (C) Snapshot of the genomic region around the *Cidea* gene locus showing both ChIP-seq replicates at d0 and d7 in BAs. (D) Heatmaps showing the level of gene expression (mRNA) and chromatin status at corresponding promoters throughout the differentiation from MSCs into BAs.
(TIF)

S3 Fig Epigenomic landscapes at *Ucp1* and *Ppara* genes during BA differentiation. **Related to Fig 1.** Snapshots of the genomic regions surrounding (A) *Ucp1* and (B) *Ppara* genes in the UCSC Genome Browser featuring a panel of chromatin marks, PPARγ binding, and mRNA expression during BA differentiation.
(TIF)

S4 Fig LncRNA expression analyses and examples of lineage specifically expressed mRNA and lncRNA. **Related to Fig 2.** (A) *Ucp1* and *Clstn3* are shown as examples of BA-specific coding genes. *Nrip1* and *Trem2* are shown as examples of WA-specific coding genes. Gene expression patterns during BA and WA differentiation, in primary BAT and WAT SVF cell derived mature adipocytes (pri-BA and pri-WA), as well as in BAT and WAT are shown. (B) Differentiation stage-specifically and lineage-specifically expressed lncRNAs during BA and WA differentiation. Lower panels illustrate the expression patterns of Blnc1 (AK038898 or NONMMUG043631), a BA specific lncRNA; and NONMMUG023147 / 8, lncRNAs predominantly expressed in mature WAs.
(TIF)

S5 Fig Stage-specific miRNAs during BA and WA differentiation. Related to Fig 2.
(TIF)

S6 Fig Enrichment of H3K27me3 and H3K4me1 at BA specific, WA specific and common adipogenic genes during BA and WA differentiation. **Related to Fig 2.** (A)-(B) Box plots showing the enrichment of (A) H3K27me3 and (B) H3K4me1 at BA specific, WA specific and common adipogenic genes during BA and WA differentiation. The table in panel (B) gives the statistical significance for the higher levels of H3K4me1 at BA specific genes in BA as compared to WAs.
(TIF)

S7 Fig *De novo* motif analysis of stage-specific enhancers during BA and WA differentiation. **Related to Fig 3.** (A) BA and (B) WA stage-specific enhancers were defined as described in Fig 3 and screened for enrichment of *de novo* motifs using HOMER. The tables list the enriched motifs ranked according to their p-values and suggested candidate TFs bound to these motifs. Candidates with matching scores >0.8 were colored in black and candidates with scores 0.7–0.8 were colored in grey.
(TIF)

S8 Fig Examples of SE-associated genes and PPARγ ChIP-seq based SE analysis in BAs. **Related to Fig 3.** (A) RNA-seq, H3K27ac and PPARγ ChIP-seq profiles at the *Fabp4* and *Ppara* genes as examples of top ranked SE-associated genes either common for BA and WA or specific for BA. The black bars indicate the location of SEs. (B) A higher percentage of SE-associated genes tends to be activated during brown adipogenesis than typical enhancer (TE) associated genes. (C) PPARγ ChIP-seq signals were used to identify SEs in BAs. Genes associated with some of the top ranked SEs were listed. (D) SE-associated genes from (C) tend to be highly upregulated during adipogenesis as compared to typical enhancer (TE) associated genes.
(TIF)

S9 Fig Overlap between SE-associated genes and BMP7 transiently activated genes in BMP7 treated C3H10T1/2 cells. **Related to Fig 4.** (A) Venn diagram showing the overlap between genes associated with a SE (468 genes) after BMP7 treatment (d0 +BMP7) and genes transiently induced by BMP7 (89 genes as shown in Fig 4A). The 14 overlapping genes are listed in the right panel. (B) Epigenomic landscape and RNA-seq tracks at the genomic region around *Fgfr3* gene before (d-3), and after treatment with (d0 +BMP7) or without (d0 -BMP7) BMP7 for 3 days. The black bar indicates the SE.
(TIF)

S10 Fig Effects of over-expression of *Six1*, *Sox13*, *Pim1* and *Rreb1* on brown marker expression, mitochondrial activity and adipogenic differentiation of SVF cells isolated from subcutaneous WAT (scWAT). **Related to Fig 5.** (A) Oil-Red-O staining of SVF cells isolated from posterior scWAT after *in vitro* differentiation for 7 days. Cells were lenti-virally transduced to over-express the indicated genes tagged with tGFP before differentiation. Cells over-expressing only tGFP served as negative control. (B) Expression of brown / mitochondrial marker genes was measured by qRT-PCR on day 7 of adipogenesis. (C) Expression of *Ucp1* gene in cells treated with or without forskolin.(D) Expression of general adipogenic genes on day 7 of adipogenesis. (E) qRT-PCR analysis confirmed the over-expression of the transduced genes using a primer pair targeting the tGFP coding sequence. (F) Measurement of oxygen consumption rates (OCR) in cells transduced with the indicated genes after 7 days of differentiation. (G) Basal respiration, proton leak, ATP production and maximal respiration were determined according to the OCR values in (F). Panels (B)-(E) summarize the average of four independent experiments. Panels (F) and (G) show the representative result of two independent biological replicates (assays performed in quadruplets). Error bars represent standard deviation. p-values (paired student’s t-test): * <0.05; **<0.01.
(TIF)

S11 Fig Effects of knock-down of *Six1*, *Sox13*, *Pim1* and *Rreb1* on brown marker expression and adipogenic differentiation of SVF cells isolated from scWAT. **Related to Fig 6.** (A) Oil-Red-O staining of SVF cells isolated from scWAT after *in vitro* differentiation for 7 days. Before adipogenic induction, cells were transfected with two independent (#1 and #2) locked nucleic acid (LNA) longRNA GapmeRs targeting the *Six1*, *Sox13*, *Pim1*, or *Rreb1* genes. Scrambled (Scr) or *Pparg*-targeting LNA GapmeRs served as negative and positive controls, respectively. (B) Expression of brown / mitochondrial marker genes was measured by qRT-PCR on day 7 of adipogenesis. (C) Expression of general adipogenic genes on day 7 of adipogenesis. (D) mRNA levels of targeted genes were assayed 24-hour post transfection. Panels (B)-(D) summarize the average of three independent experiments. Error bars represent standard deviation. *p*-values (paired student’s t-test): * <0.05; ** <0.01.
(TIF)

S12 Fig SIX1 binds to an upstream enhancer element of the *Cidea* gene and promotes its expression. **Related to Fig 7.** Enlarged view of the upstream region of the *Cidea* gene reveals the existence of an enhancer element bound by SIX1, PPARγ and marked by H3K27ac. A 222bp SIX1-bound region was PCR-amplified, cloned and tested for its regulatory potential using luciferase assay. SIX1 binding motif (TCAGGTTTC) was highlighted in bold letters.
(TIF)

S1 Table Pearson correlation coefficients of RNA-seq and ChIP-seq datasets. (XLSX)

S2 Table Gene expression and GO analysis of coding genes, lncRNAs and miRNAs. (XLSX)

S3 Table Enrichment of SIX1 binding motif at H3K27ac marked enhancers is significantly higher in BAT than in WAT. (XLSX)

S4 Table Super-enhancers and super-enhancer-associated genes defined by PPARγ binding and H3K27ac. (XLSX)

S5 Table BMP7 responsive genes. (XLSX)

S6 Table List of antibodies and primers. (XLSX)

## References

[1] Wing RR, Phelan S. Long-term weight loss maintenance. The American journal of clinical nutrition. 2005;82(1 Suppl):222S–5S. . 16002825 10.1093/ajcn/82.1.222S

[2] van Marken Lichtenbelt WD, Vanhommerig JW, Smulders NM, Drossaerts JM, Kemerink GJ, Bouvy ND, et al Cold-activated brown adipose tissue in healthy men. The New England journal of medicine. 2009;360(15):1500–8. 10.1056/NEJMoa0808718 19357405

[3] Cypess AM, Lehman S, Williams G, Tal I, Rodman D, Goldfine AB, et al Identification and importance of brown adipose tissue in adult humans. The New England journal of medicine. 2009;360(15):1509–17. PubMed Central PMCID: PMC2859951. 10.1056/NEJMoa0810780 19357406 PMC2859951

[4] Virtanen KA, Lidell ME, Orava J, Heglind M, Westergren R, Niemi T, et al Functional brown adipose tissue in healthy adults. The New England journal of medicine. 2009;360(15):1518–25. 10.1056/NEJMoa0808949 19357407

[5] Saito M, Okamatsu-Ogura Y, Matsushita M, Watanabe K, Yoneshiro T, Nio-Kobayashi J, et al High incidence of metabolically active brown adipose tissue in healthy adult humans: effects of cold exposure and adiposity. Diabetes. 2009;58(7):1526–31. PubMed Central PMCID: PMC2699872. 10.2337/db09-0530 19401428 PMC2699872

[6] Harms M, Seale P. Brown and beige fat: development, function and therapeutic potential. Nat Med. 2013;19(10):1252–63. 10.1038/nm.3361 24100998

[7] Schulz TJ, Tseng YH. Brown adipose tissue: development, metabolism and beyond. The Biochemical journal. 2013;453(2):167–78. Epub 2013/06/29. 10.1042/BJ20130457 23805974 PMC3887508

[8] Bartelt A, Bruns OT, Reimer R, Hohenberg H, Ittrich H, Peldschus K, et al Brown adipose tissue activity controls triglyceride clearance. Nat Med. 2011;17(2):200–5. 10.1038/nm.2297 21258337

[9] Wu J, Cohen P, Spiegelman BM. Adaptive thermogenesis in adipocytes: is beige the new brown? Genes Dev. 2013;27(3):234–50. Epub 2013/02/08. PubMed Central PMCID: PMC3576510. 10.1101/gad.211649.112 23388824 PMC3576510

[10] Loft A, Forss I, Siersbaek MS, Schmidt SF, Larsen AS, Madsen JG, et al Browning of human adipocytes requires KLF11 and reprogramming of PPARgamma superenhancers. Genes Dev. 2015;29(1):7–22. PubMed Central PMCID: PMCPMC4281566. 10.1101/gad.250829.114 25504365 PMC4281566

[11] Zhao XY, Li S, Wang GX, Yu Q, Lin JD. A long noncoding RNA transcriptional regulatory circuit drives thermogenic adipocyte differentiation. Mol Cell. 2014;55(3):372–82. PubMed Central PMCID: PMC4127104. 10.1016/j.molcel.2014.06.004 25002143 PMC4127104

[12] Alvarez-Dominguez JR, Bai Z, Xu D, Yuan B, Lo KA, Yoon MJ, et al De Novo Reconstruction of Adipose Tissue Transcriptomes Reveals Long Non-coding RNA Regulators of Brown Adipocyte Development. Cell Metab. 2015;21(5):764–76. PubMed Central PMCID: PMC4429916. 10.1016/j.cmet.2015.04.003 25921091 PMC4429916

[13] Sun L, Xie H, Mori MA, Alexander R, Yuan B, Hattangadi SM, et al Mir193b-365 is essential for brown fat differentiation. Nat Cell Biol. 2011;13(8):958–65. 10.1038/ncb2286 21743466 PMC3149720

[14] Whyte WA, Orlando DA, Hnisz D, Abraham BJ, Lin CY, Kagey MH, et al Master transcription factors and mediator establish super-enhancers at key cell identity genes. Cell. 2013;153(2):307–19. PubMed Central PMCID: PMC3653129. 10.1016/j.cell.2013.03.035 23582322 PMC3653129

[15] Mikkelsen TS, Xu Z, Zhang X, Wang L, Gimble JM, Lander ES, et al Comparative epigenomic analysis of murine and human adipogenesis. Cell. 2010;143(1):156–69. PubMed Central PMCID: PMC2950833. 10.1016/j.cell.2010.09.006 20887899 PMC2950833

[16] Rajakumari S, Wu J, Ishibashi J, Lim HW, Giang AH, Won KJ, et al EBF2 determines and maintains brown adipocyte identity. Cell Metab. 2013;17(4):562–74. PubMed Central PMCID: PMC3622114. 10.1016/j.cmet.2013.01.015 23499423 PMC3622114

[17] Harms MJ, Lim HW, Ho Y, Shapira SN, Ishibashi J, Rajakumari S, et al PRDM16 binds MED1 and controls chromatin architecture to determine a brown fat transcriptional program. Genes Dev. 2015;29(3):298–307. PubMed Central PMCID: PMC4318146. 10.1101/gad.252734.114 25644604 PMC4318146

[18] Santos-Rosa H, Schneider R, Bannister AJ, Sherriff J, Bernstein BE, Emre NC, et al Active genes are tri-methylated at K4 of histone H3. Nature. 2002;419(6905):407–11. 10.1038/nature01080 12353038

[19] Li B, Carey M, Workman JL. The role of chromatin during transcription. Cell. 2007;128(4):707–19. 10.1016/j.cell.2007.01.015 17320508

[20] Lachner M, Jenuwein T. The many faces of histone lysine methylation. Curr Opin Cell Biol. 2002;14(3):286–98. . 12067650 10.1016/s0955-0674(02)00335-6

[21] Heintzman ND, Stuart RK, Hon G, Fu Y, Ching CW, Hawkins RD, et al Distinct and predictive chromatin signatures of transcriptional promoters and enhancers in the human genome. Nature genetics. 2007;39(3):311–8. 10.1038/ng1966 17277777

[22] Bernstein BE, Mikkelsen TS, Xie X, Kamal M, Huebert DJ, Cuff J, et al A bivalent chromatin structure marks key developmental genes in embryonic stem cells. Cell. 2006;125(2):315–26. 10.1016/j.cell.2006.02.041 16630819

[23] Steger DJ, Grant GR, Schupp M, Tomaru T, Lefterova MI, Schug J, et al Propagation of adipogenic signals through an epigenomic transition state. Genes Dev. 2010;24(10):1035–44. PubMed Central PMCID: PMCPMC2867208. 10.1101/gad.1907110 20478996 PMC2867208

[24] Tseng YH, Kokkotou E, Schulz TJ, Huang TL, Winnay JN, Taniguchi CM, et al New role of bone morphogenetic protein 7 in brown adipogenesis and energy expenditure. Nature. 2008;454(7207):1000–4. PubMed Central PMCID: PMC2745972. 10.1038/nature07221 18719589 PMC2745972

[25] Lane MD, Tang QQ, Jiang MS. Role of the CCAAT enhancer binding proteins (C/EBPs) in adipocyte differentiation. Biochem Biophys Res Commun. 1999;266(3):677–83. Epub 1999/12/22. 10.1006/bbrc.1999.1885 10603305

[26] Xie C, Yuan J, Li H, Li M, Zhao G, Bu D, et al NONCODEv4: exploring the world of long non-coding RNA genes. Nucleic acids research. 2014;42(Database issue):D98–103. PubMed Central PMCID: PMC3965073. 10.1093/nar/gkt1222 24285305 PMC3965073

[27] Sun L, Goff LA, Trapnell C, Alexander R, Lo KA, Hacisuleyman E, et al Long noncoding RNAs regulate adipogenesis. Proc Natl Acad Sci U S A. 2013;110(9):3387–92. PubMed Central PMCID: PMC3587215. 10.1073/pnas.1222643110 23401553 PMC3587215

[28] Ussar S, Lee KY, Dankel SN, Boucher J, Haering MF, Kleinridders A, et al ASC-1, PAT2, and P2RX5 are cell surface markers for white, beige, and brown adipocytes. Science translational medicine. 2014;6(247):247ra103 PubMed Central PMCID: PMC4356008. 10.1126/scitranslmed.3008490 25080478 PMC4356008

[29] Leung CO, Wong CC, Fan DN, Kai AK, Tung EK, Xu IM, et al PIM1 regulates glycolysis and promotes tumor progression in hepatocellular carcinoma. Oncotarget. 2015;6(13):10880–92. 10.18632/oncotarget.3534 25834102 PMC4484426

[30] Beharry Z, Mahajan S, Zemskova M, Lin YW, Tholanikunnel BG, Xia Z, et al The Pim protein kinases regulate energy metabolism and cell growth. Proc Natl Acad Sci U S A. 2011;108(2):528–33. PubMed Central PMCID: PMC3021022. 10.1073/pnas.1013214108 21187426 PMC3021022

[31] Nautiyal J, Christian M, Parker MG. Distinct functions for RIP140 in development, inflammation, and metabolism. Trends in endocrinology and metabolism: TEM. 2013;24(9):451–9. 10.1016/j.tem.2013.05.001 23742741

[32] Pan D, Huang L, Zhu LJ, Zou T, Ou J, Zhou W, et al Jmjd3-Mediated H3K27me3 Dynamics Orchestrate Brown Fat Development and Regulate White Fat Plasticity. Dev Cell. 2015;35(5):568–83. PubMed Central PMCID: PMCPMC4679478. 10.1016/j.devcel.2015.11.002 26625958 PMC4679478

[33] Creyghton MP, Cheng AW, Welstead GG, Kooistra T, Carey BW, Steine EJ, et al Histone H3K27ac separates active from poised enhancers and predicts developmental state. Proc Natl Acad Sci U S A. 2010;107(50):21931–6. Epub 2010/11/26. PubMed Central PMCID: PMC3003124. 10.1073/pnas.1016071107 21106759 PMC3003124

[34] Heinz S, Benner C, Spann N, Bertolino E, Lin YC, Laslo P, et al Simple combinations of lineage-determining transcription factors prime cis-regulatory elements required for macrophage and B cell identities. Mol Cell. 2010;38(4):576–89. PubMed Central PMCID: PMC2898526. 10.1016/j.molcel.2010.05.004 20513432 PMC2898526

[35] Yue F, Cheng Y, Breschi A, Vierstra J, Wu W, Ryba T, et al A comparative encyclopedia of DNA elements in the mouse genome. Nature. 2014;515(7527):355–64. PubMed Central PMCID: PMCPMC4266106. 10.1038/nature13992 25409824 PMC4266106

[36] Hnisz D, Abraham BJ, Lee TI, Lau A, Saint-Andre V, Sigova AA, et al Super-enhancers in the control of cell identity and disease. Cell. 2013;155(4):934–47. PubMed Central PMCID: PMC3841062. 10.1016/j.cell.2013.09.053 24119843 PMC3841062

[37] Liu CT, Monda KL, Taylor KC, Lange L, Demerath EW, Palmas W, et al Genome-wide association of body fat distribution in African ancestry populations suggests new loci. PLoS genetics. 2013;9(8):e1003681 PubMed Central PMCID: PMC3744443. 10.1371/journal.pgen.1003681 23966867 PMC3744443

[38] Replication DIG, Meta-analysis C, Asian Genetic Epidemiology Network Type 2 Diabetes C, South Asian Type 2 Diabetes C, Mexican American Type 2 Diabetes C, Type 2 Diabetes Genetic Exploration by Nex-generation sequencing in muylti-Ethnic Samples C, et al Genome-wide trans-ancestry meta-analysis provides insight into the genetic architecture of type 2 diabetes susceptibility. Nature genetics. 2014;46(3):234–44. PubMed Central PMCID: PMC3969612. 10.1038/ng.2897 24509480 PMC3969612

[39] Scott RA, Lagou V, Welch RP, Wheeler E, Montasser ME, Luan J, et al Large-scale association analyses identify new loci influencing glycemic traits and provide insight into the underlying biological pathways. Nature genetics. 2012;44(9):991–1005. PubMed Central PMCID: PMC3433394. 10.1038/ng.2385 22885924 PMC3433394

[40] Kamachi Y, Kondoh H. Sox proteins: regulators of cell fate specification and differentiation. Development. 2013;140(20):4129–44. 10.1242/dev.091793 24086078

[41] Stockl S, Bauer RJ, Bosserhoff AK, Gottl C, Grifka J, Grassel S. Sox9 modulates cell survival and adipogenic differentiation of multipotent adult rat mesenchymal stem cells. Journal of cell science. 2013;126(Pt 13):2890–902. 10.1242/jcs.124305 23606745

[42] Cao W, Daniel KW, Robidoux J, Puigserver P, Medvedev AV, Bai X, et al p38 mitogen-activated protein kinase is the central regulator of cyclic AMP-dependent transcription of the brown fat uncoupling protein 1 gene. Molecular and cellular biology. 2004;24(7):3057–67. 10.1128/MCB.24.7.3057-3067.2004 15024092 PMC371122

[43] Fisher FM, Kleiner S, Douris N, Fox EC, Mepani RJ, Verdeguer F, et al FGF21 regulates PGC-1alpha and browning of white adipose tissues in adaptive thermogenesis. Genes Dev. 2012;26(3):271–81. PubMed Central PMCID: PMCPMC3278894. 10.1101/gad.177857.111 22302939 PMC3278894

[44] Seale P, Conroe HM, Estall J, Kajimura S, Frontini A, Ishibashi J, et al Prdm16 determines the thermogenic program of subcutaneous white adipose tissue in mice. The Journal of clinical investigation. 2011;121(1):96–105. Epub 2010/12/03. PubMed Central PMCID: PMC3007155. 10.1172/JCI44271 21123942 PMC3007155

[45] Lee TI, Young RA. Transcription of eukaryotic protein-coding genes. Annu Rev Genet. 2000;34:77–137. 10.1146/annurev.genet.34.1.77 11092823

[46] Lee YH, Petkova AP, Mottillo EP, Granneman JG. In vivo identification of bipotential adipocyte progenitors recruited by beta3-adrenoceptor activation and high-fat feeding. Cell Metab. 2012;15(4):480–91. PubMed Central PMCID: PMC3322390. 10.1016/j.cmet.2012.03.009 22482730 PMC3322390

[47] Rosenwald M, Perdikari A, Rulicke T, Wolfrum C. Bi-directional interconversion of brite and white adipocytes. Nat Cell Biol. 2013;15(6):659–67. 10.1038/ncb2740 23624403

[48] Wang QA, Tao C, Gupta RK, Scherer PE. Tracking adipogenesis during white adipose tissue development, expansion and regeneration. Nat Med. 2013;19(10):1338–44. PubMed Central PMCID: PMC4075943. 10.1038/nm.3324 23995282 PMC4075943

[49] Siersbaek MS, Loft A, Aagaard MM, Nielsen R, Schmidt SF, Petrovic N, et al Genome-wide profiling of peroxisome proliferator-activated receptor gamma in primary epididymal, inguinal, and brown adipocytes reveals depot-selective binding correlated with gene expression. Mol Cell Biol. 2012;32(17):3452–63. PubMed Central PMCID: PMCPMC3421998. 10.1128/MCB.00526-12 22733994 PMC3421998

[50] Siersbaek R, Baek S, Rabiee A, Nielsen R, Traynor S, Clark N, et al Molecular architecture of transcription factor hotspots in early adipogenesis. Cell Rep. 2014;7(5):1434–42. 10.1016/j.celrep.2014.04.043 24857666 PMC6360525

[51] Siersbaek R, Nielsen R, John S, Sung MH, Baek S, Loft A, et al Extensive chromatin remodelling and establishment of transcription factor 'hotspots' during early adipogenesis. EMBO J. 2011;30(8):1459–72. PubMed Central PMCID: PMCPMC3102274. 10.1038/emboj.2011.65 21427703 PMC3102274

[52] Siersbaek R, Rabiee A, Nielsen R, Sidoli S, Traynor S, Loft A, et al Transcription factor cooperativity in early adipogenic hotspots and super-enhancers. Cell Rep. 2014;7(5):1443–55. 10.1016/j.celrep.2014.04.042 24857652

[53] Lefterova MI, Zhang Y, Steger DJ, Schupp M, Schug J, Cristancho A, et al PPARgamma and C/EBP factors orchestrate adipocyte biology via adjacent binding on a genome-wide scale. Genes Dev. 2008;22(21):2941–52. PubMed Central PMCID: PMCPMC2577797. 10.1101/gad.1709008 18981473 PMC2577797

[54] Zhang Q, Ramlee MK, Brunmeir R, Villanueva CJ, Halperin D, Xu F. Dynamic and distinct histone modifications modulate the expression of key adipogenesis regulatory genes. Cell Cycle. 2012;11(23):4310–22. PubMed Central PMCID: PMC3552913. 10.4161/cc.22224 23085542 PMC3552913

[55] Peng X, Wu J, Brunmeir R, Kim SY, Zhang Q, Ding C, et al TELP, a sensitive and versatile library construction method for next-generation sequencing. Nucleic acids research. 2015;43(6):e35 PubMed Central PMCID: PMC4381050. 10.1093/nar/gku818 25223787 PMC4381050

[56] Zhang Y, Liu T, Meyer CA, Eeckhoute J, Johnson DS, Bernstein BE, et al Model-based analysis of ChIP-Seq (MACS). Genome biology. 2008;9(9):R137 PubMed Central PMCID: PMC2592715. 10.1186/gb-2008-9-9-r137 18798982 PMC2592715

[57] Trapnell C, Roberts A, Goff L, Pertea G, Kim D, Kelley DR, et al Differential gene and transcript expression analysis of RNA-seq experiments with TopHat and Cufflinks. Nature protocols. 2012;7(3):562–78. PubMed Central PMCID: PMC3334321. 10.1038/nprot.2012.016 22383036 PMC3334321

[58] Barrera LO, Li Z, Smith AD, Arden KC, Cavenee WK, Zhang MQ, et al Genome-wide mapping and analysis of active promoters in mouse embryonic stem cells and adult organs. Genome Res. 2008;18(1):46–59. PubMed Central PMCID: PMC2134779. 10.1101/gr.6654808 18042645 PMC2134779

[59] Schug J, Schuller WP, Kappen C, Salbaum JM, Bucan M, Stoeckert CJ Jr. Promoter features related to tissue specificity as measured by Shannon entropy. Genome biology. 2005;6(4):R33 PubMed Central PMCID: PMC1088961. 10.1186/gb-2005-6-4-r33 15833120 PMC1088961

[60] Shen Y, Yue F, McCleary DF, Ye Z, Edsall L, Kuan S, et al A map of the cis-regulatory sequences in the mouse genome. Nature. 2012;488(7409):116–20. PubMed Central PMCID: PMC4041622. 10.1038/nature11243 22763441 PMC4041622

[61] Xie W, Schultz MD, Lister R, Hou Z, Rajagopal N, Ray P, et al Epigenomic analysis of multilineage differentiation of human embryonic stem cells. Cell. 2013;153(5):1134–48. PubMed Central PMCID: PMC3786220. 10.1016/j.cell.2013.04.022 23664764 PMC3786220

[62] Dennis G Jr., Sherman BT, Hosack DA, Yang J, Gao W, Lane HC, et al DAVID: Database for Annotation, Visualization, and Integrated Discovery. Genome biology. 2003;4(5):P3 . 12734009 PMC193660

[63] McLean CY, Bristor D, Hiller M, Clarke SL, Schaar BT, Lowe CB, et al GREAT improves functional interpretation of cis-regulatory regions. Nature biotechnology. 2010;28(5):495–501. 10.1038/nbt.1630 20436461 PMC4840234

