# Supplementary figures and images for: Comparative Transcriptomic and Epigenomic Analyses Reveal New Regulators of Murine Brown Adipogenesis
Source: PLoS Genet. 2016 Dec 6;12(12):e1006474. doi: 10.1371/journal.pgen.1006474 (PMC5140063; doi:10.1371/journal.pgen.1006474)

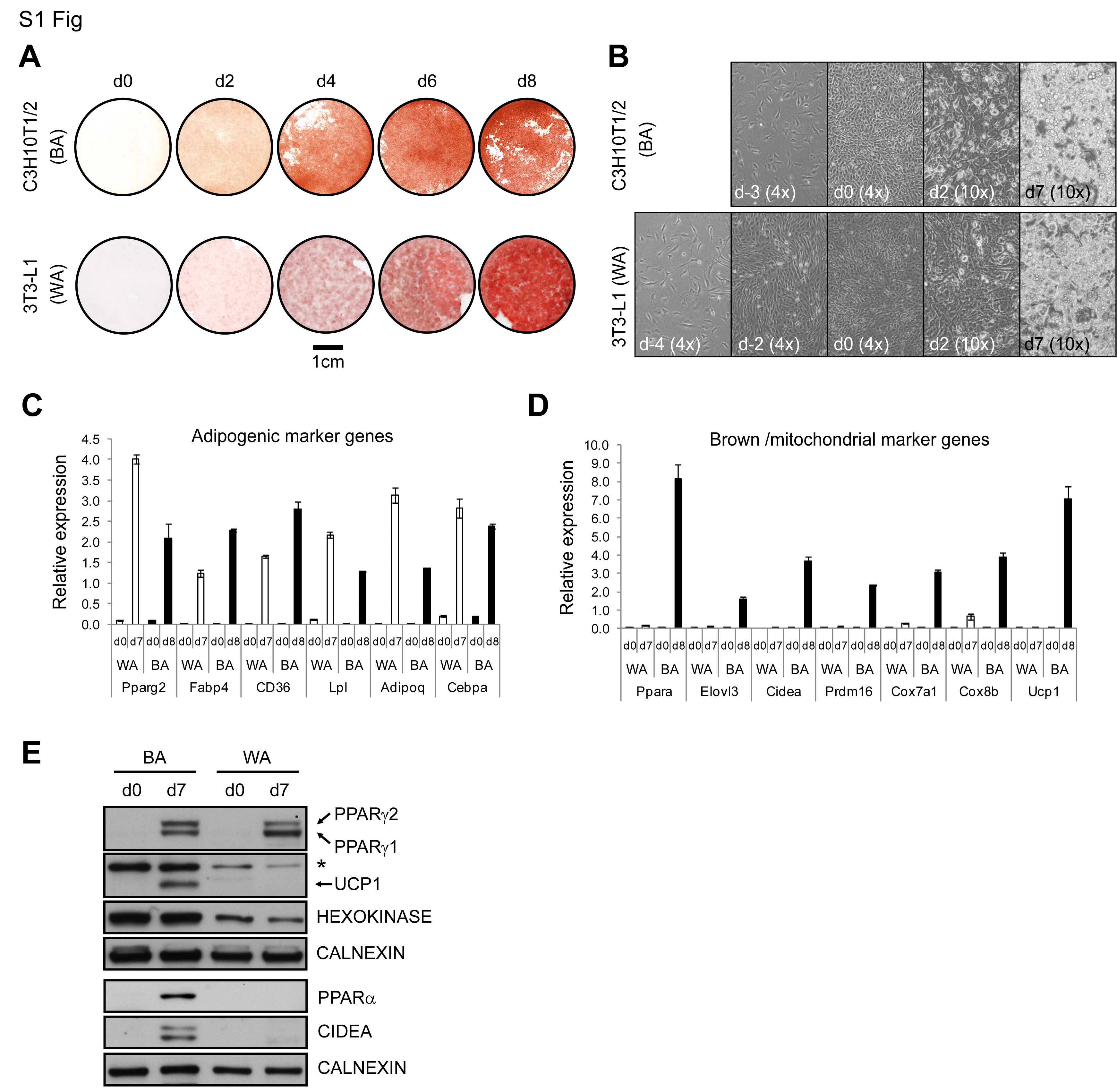

Supplement: S1 Fig — Related to Fig 1. (A) Oil-Red-O staining of the cells at various differentiation stages during C3H10T1/2 (BA) and 3T3-L1 (WA) adipogenesis. (B) Morphology of C3H10T1/2 and 3T3-L1 cells at key stages of BA and WA differentiation. (C)-(D) Expression of (C) general adipogenic and (D) brown / mitochondrial marker genes before (d0) and after (d7/d8) differentiation. Data were normalized to Peptidylprolyl Isomerase A (Ppia) expression; graphs summarize the results of 3 technical replicates, error bars represent standard deviations. (E) Western blot confirmed the expression of PPARγ protein in mature BA and WA cells, whereas UCP1, PPARα, and CIDEA proteins were only detected in mature BAs. Hexokinase was used as a control for mitochondrial content. Calnexin served as a loading control. The asterisk (*) marks an unspecific band in the UCP1 blot. (TIF) [file pgen.1006474.s001.tif]

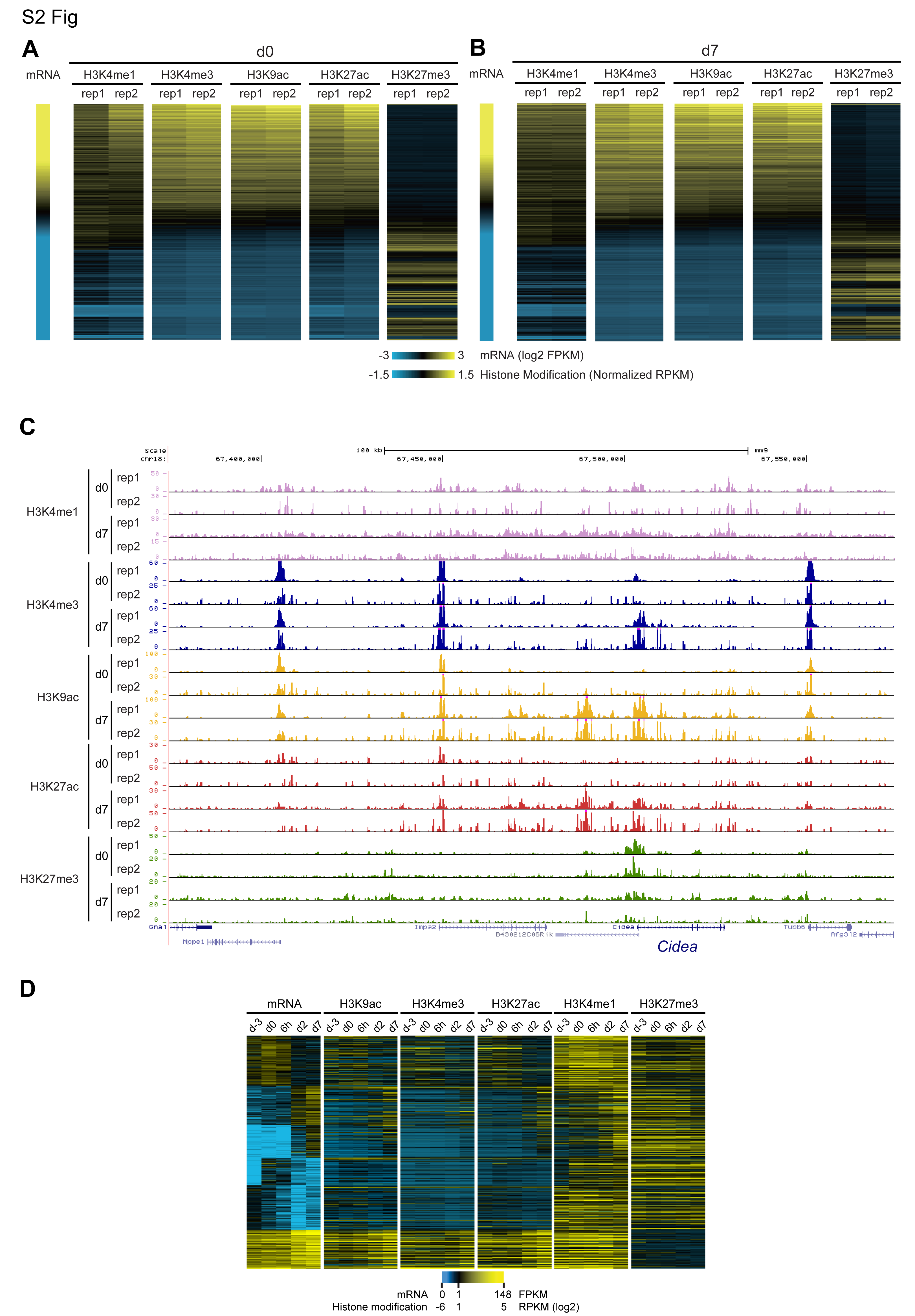

Supplement: S2 Fig — Related to Fig 1. (A)-(B) Heatmaps of H3K4me1, H3K4me3, H3K9ac, H3K27ac and H3K27me3 ChIP-seq signals at the promoter region of annotated genes at (A) d0 and (B) d7 in BAs. Note that the two independent replicates (rep1 and rep2) are highly consistent. (C) Snapshot of the genomic region around the Cidea gene locus showing both ChIP-seq replicates at d0 and d7 in BAs. (D) Heatmaps showing the level of gene expression (mRNA) and chromatin status at corresponding promoters throughout the differentiation from MSCs into BAs. (TIF) [file pgen.1006474.s002.tif]

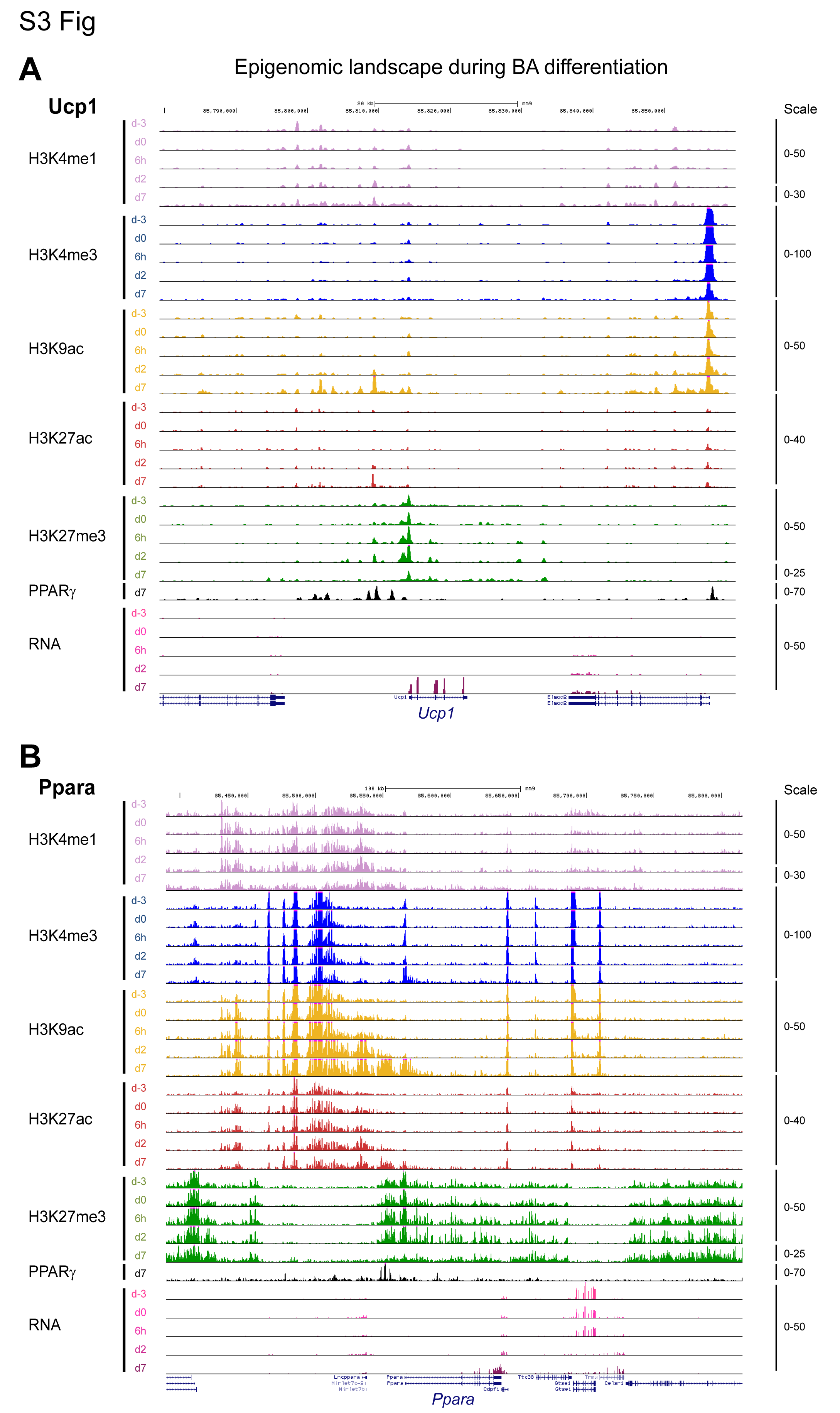

Supplement: S3 Fig — Related to Fig 1. Snapshots of the genomic regions surrounding (A) Ucp1 and (B) Ppara genes in the UCSC Genome Browser featuring a panel of chromatin marks, PPARγ binding, and mRNA expression during BA differentiation. (TIF) [file pgen.1006474.s003.tif]

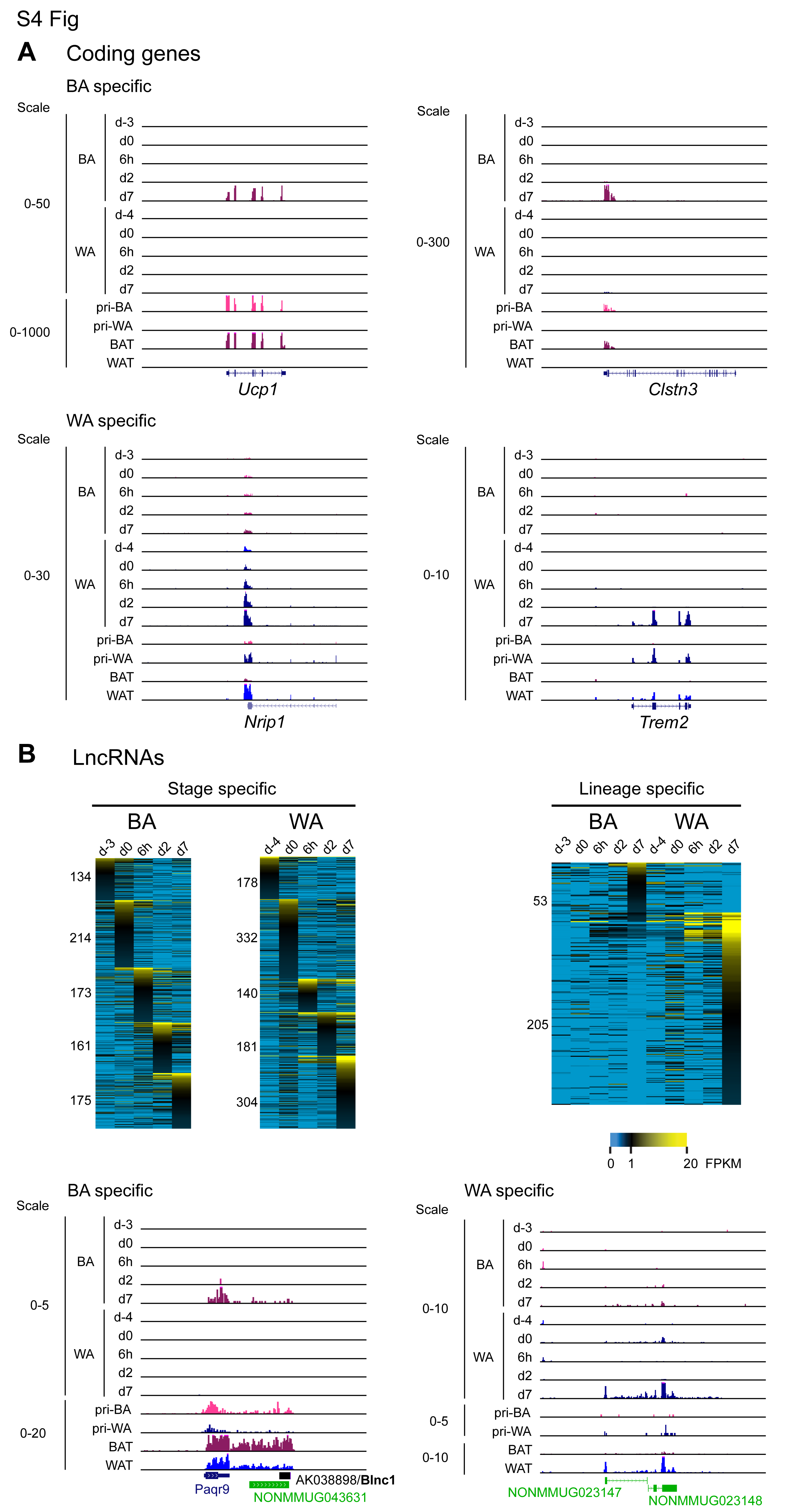

Supplement: S4 Fig — Related to Fig 2. (A) Ucp1 and Clstn3 are shown as examples of BA-specific coding genes. Nrip1 and Trem2 are shown as examples of WA-specific coding genes. Gene expression patterns during BA and WA differentiation, in primary BAT and WAT SVF cell derived mature adipocytes (pri-BA and pri-WA), as well as in BAT and WAT are shown. (B) Differentiation stage-specifically and lineage-specifically expressed lncRNAs during BA and WA differentiation. Lower panels illustrate the expression patterns of Blnc1 (AK038898 or NONMMUG043631), a BA specific lncRNA; and NONMMUG023147 / 8, lncRNAs predominantly expressed in mature WAs. (TIF) [file pgen.1006474.s004.tif]

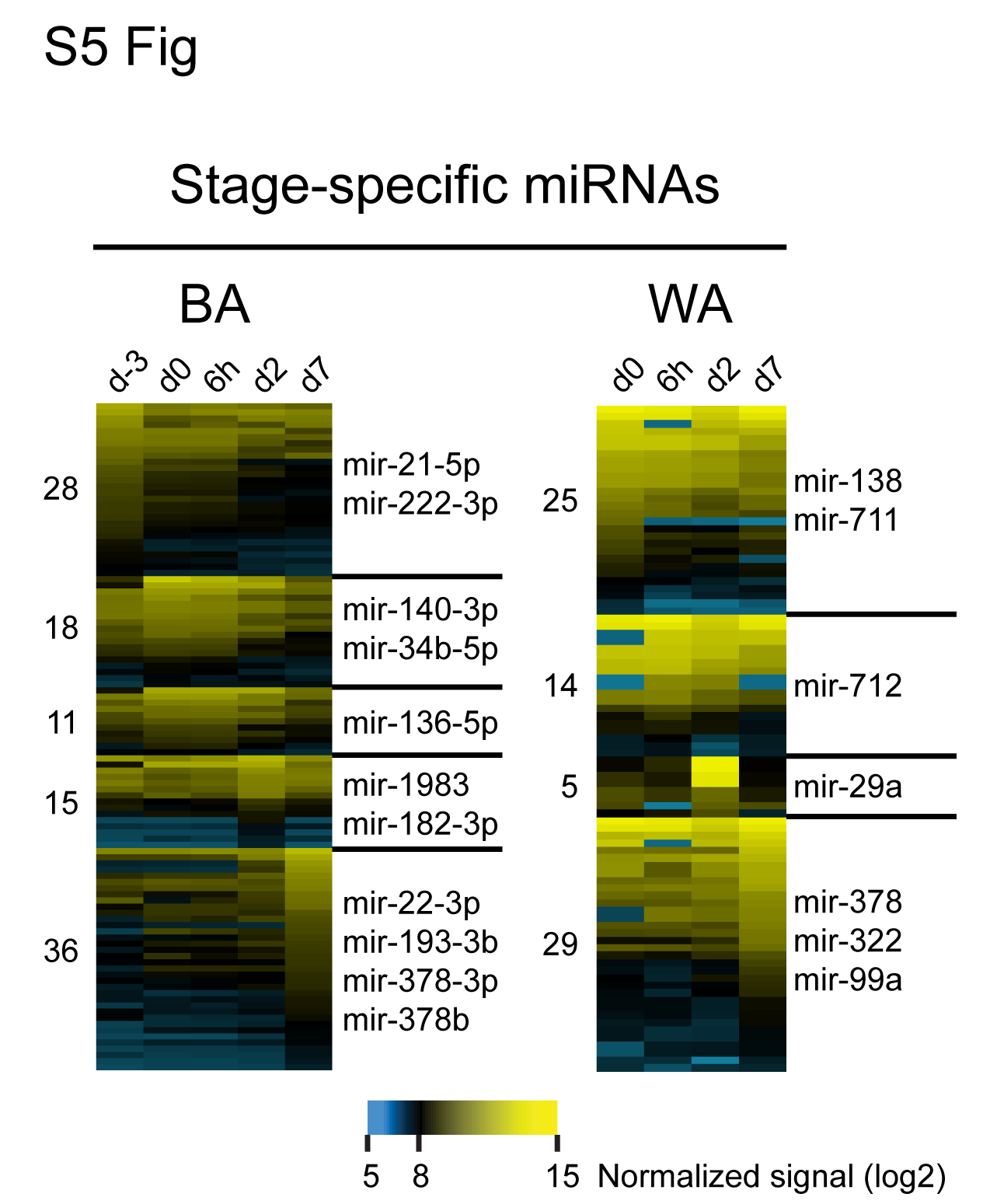

Supplement: S5 Fig — Related to Fig 2. (TIF) [file pgen.1006474.s005.tif]

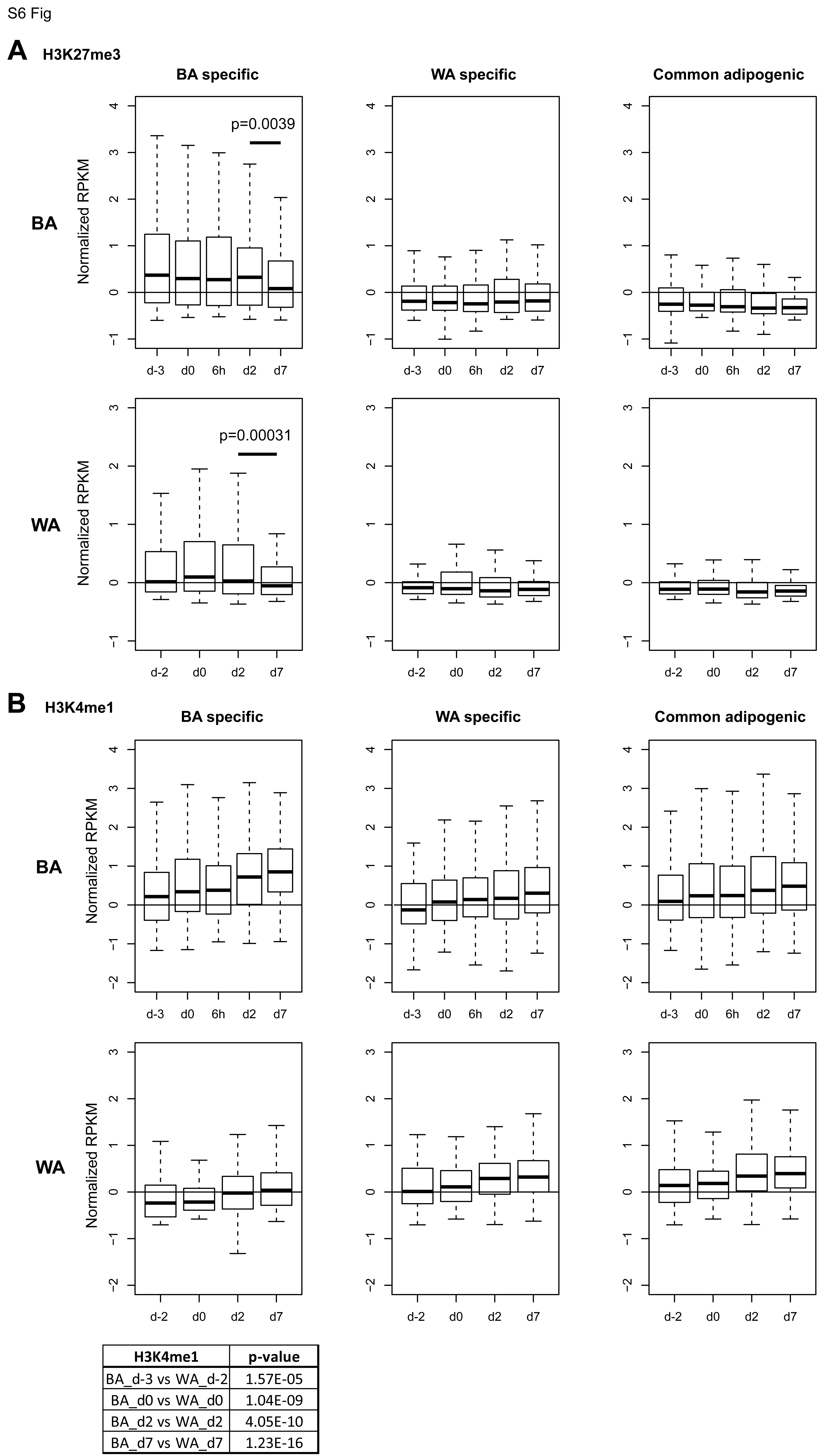

Supplement: S6 Fig — Related to Fig 2. (A)-(B) Box plots showing the enrichment of (A) H3K27me3 and (B) H3K4me1 at BA specific, WA specific and common adipogenic genes during BA and WA differentiation. The table in panel (B) gives the statistical significance for the higher levels of H3K4me1 at BA specific genes in BA as compared to WAs. (TIF) [file pgen.1006474.s006.tif]

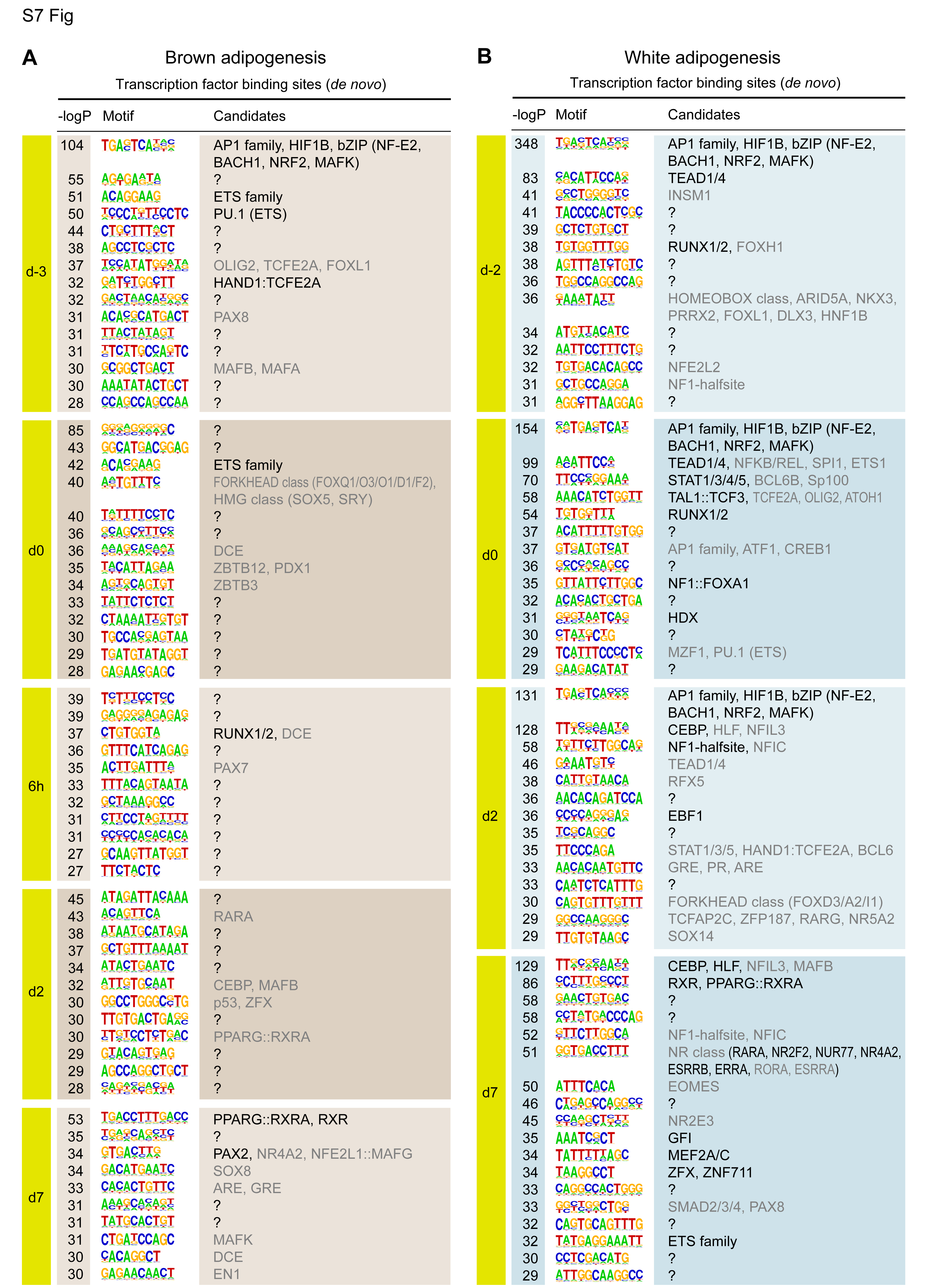

Supplement: S7 Fig — Related to Fig 3. (A) BA and (B) WA stage-specific enhancers were defined as described in Fig 3 and screened for enrichment of de novo motifs using HOMER. The tables list the enriched motifs ranked according to their p-values and suggested candidate TFs bound to these motifs. Candidates with matching scores >0.8 were colored in black and candidates with scores 0.7–0.8 were colored in grey. (TIF) [file pgen.1006474.s007.tif]

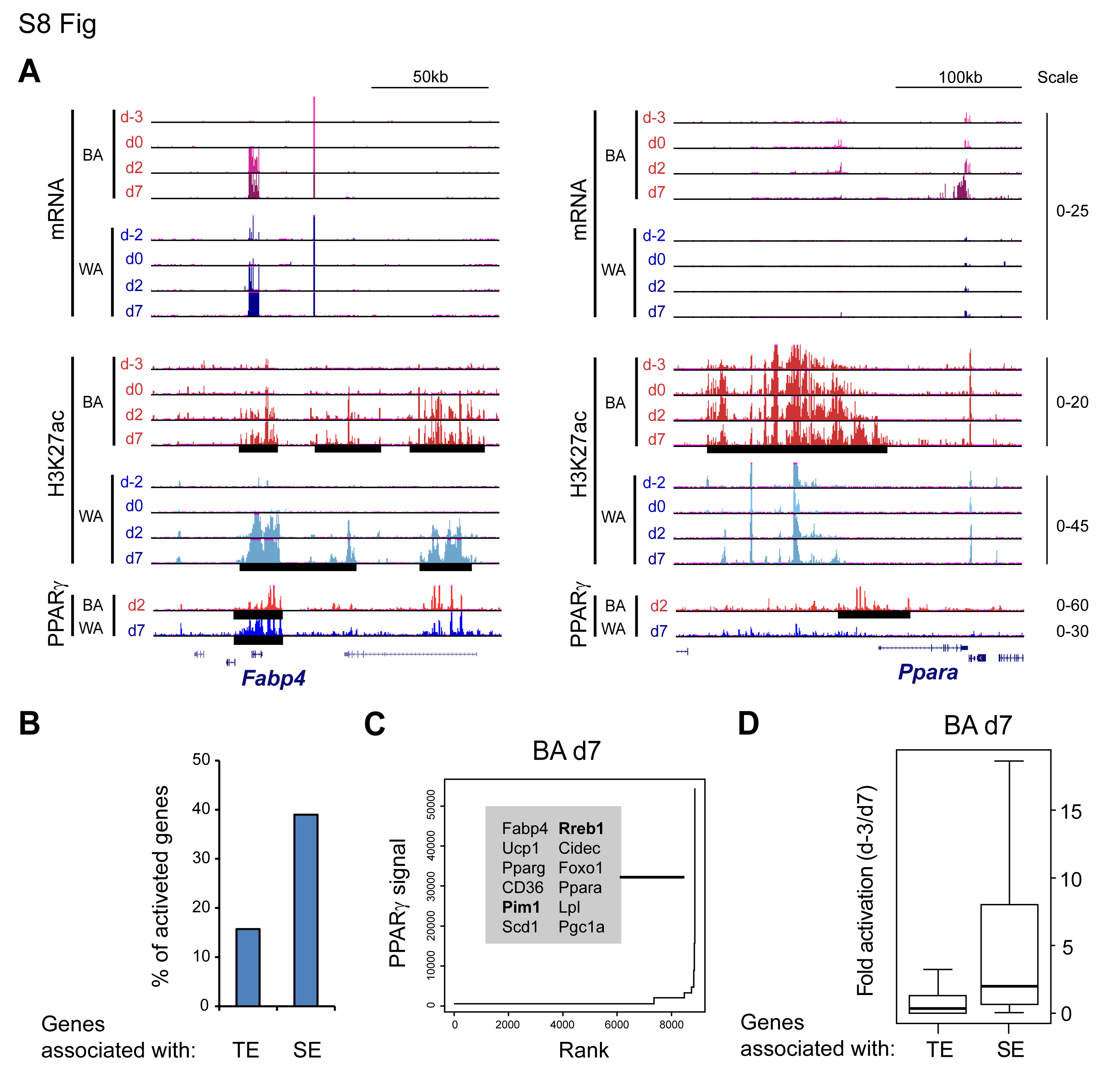

Supplement: S8 Fig — Related to Fig 3. (A) RNA-seq, H3K27ac and PPARγ ChIP-seq profiles at the Fabp4 and Ppara genes as examples of top ranked SE-associated genes either common for BA and WA or specific for BA. The black bars indicate the location of SEs. (B) A higher percentage of SE-associated genes tends to be activated during brown adipogenesis than typical enhancer (TE) associated genes. (C) PPARγ ChIP-seq signals were used to identify SEs in BAs. Genes associated with some of the top ranked SEs were listed. (D) SE-associated genes from (C) tend to be highly upregulated during adipogenesis as compared to typical enhancer (TE) associated genes. (TIF) [file pgen.1006474.s008.tif]

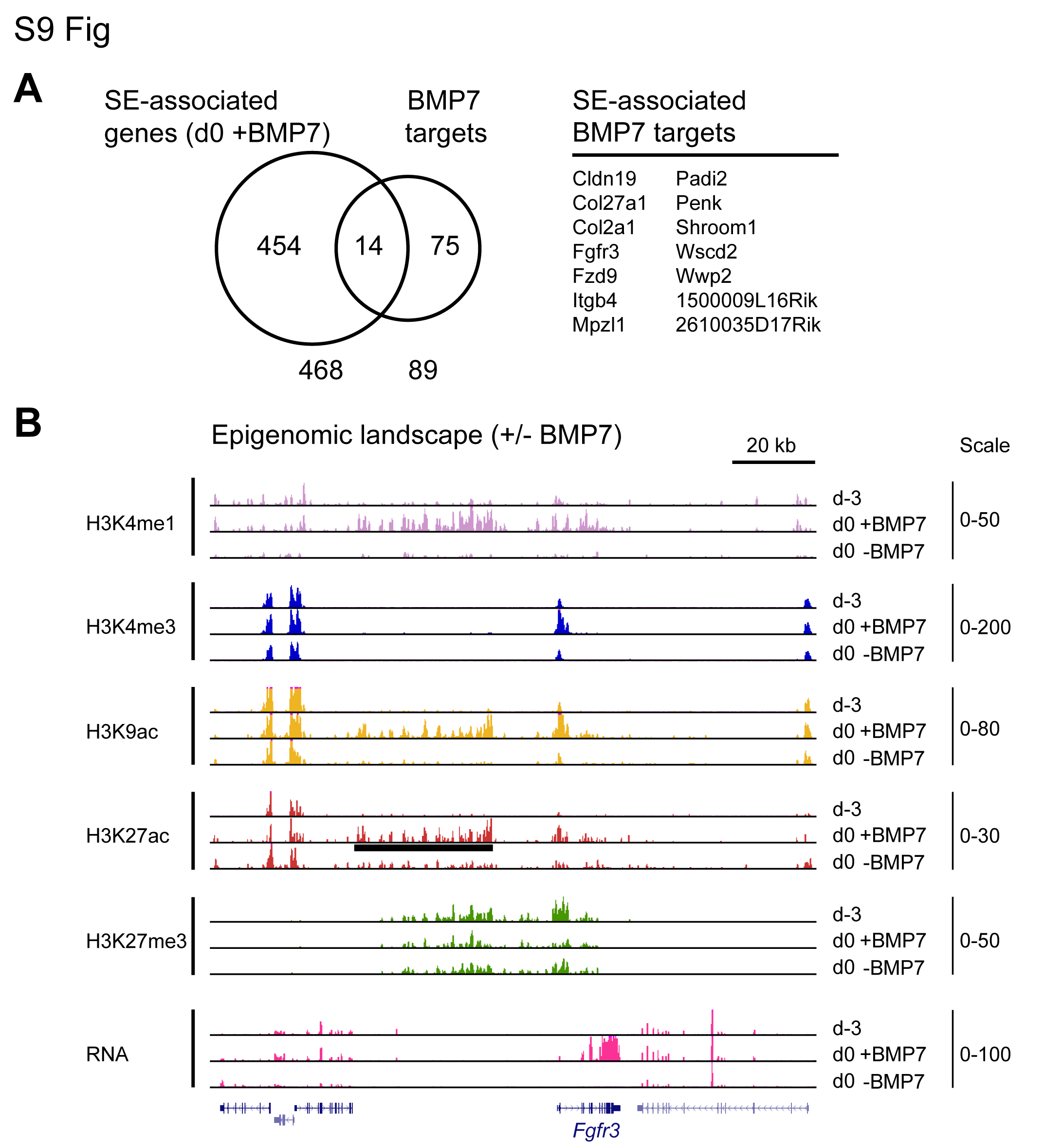

Supplement: S9 Fig — Related to Fig 4. (A) Venn diagram showing the overlap between genes associated with a SE (468 genes) after BMP7 treatment (d0 +BMP7) and genes transiently induced by BMP7 (89 genes as shown in Fig 4A). The 14 overlapping genes are listed in the right panel. (B) Epigenomic landscape and RNA-seq tracks at the genomic region around Fgfr3 gene before (d-3), and after treatment with (d0 +BMP7) or without (d0 -BMP7) BMP7 for 3 days. The black bar indicates the SE. (TIF) [file pgen.1006474.s009.tif]

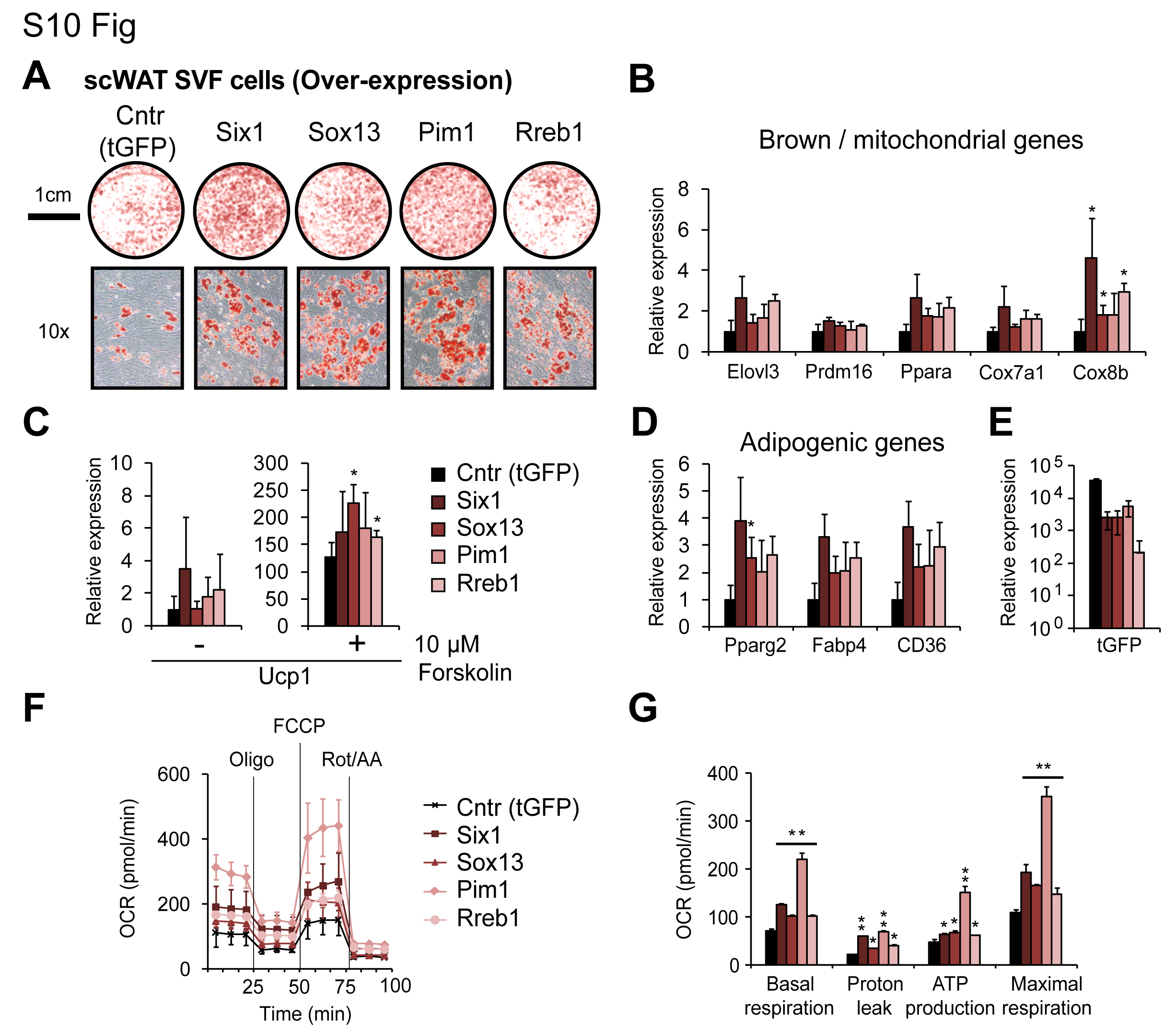

Supplement: S10 Fig — Related to Fig 5. (A) Oil-Red-O staining of SVF cells isolated from posterior scWAT after in vitro differentiation for 7 days. Cells were lenti-virally transduced to over-express the indicated genes tagged with tGFP before differentiation. Cells over-expressing only tGFP served as negative control. (B) Expression of brown / mitochondrial marker genes was measured by qRT-PCR on day 7 of adipogenesis. (C) Expression of Ucp1 gene in cells treated with or without forskolin.(D) Expression of general adipogenic genes on day 7 of adipogenesis. (E) qRT-PCR analysis confirmed the over-expression of the transduced genes using a primer pair targeting the tGFP coding sequence. (F) Measurement of oxygen consumption rates (OCR) in cells transduced with the indicated genes after 7 days of differentiation. (G) Basal respiration, proton leak, ATP production and maximal respiration were determined according to the OCR values in (F). Panels (B)-(E) summarize the average of four independent experiments. Panels (F) and (G) show the representative result of two independent biological replicates (assays performed in quadruplets). Error bars represent standard deviation. p-values (paired student’s t-test): * <0.05; **<0.01. (TIF) [file pgen.1006474.s010.tif]

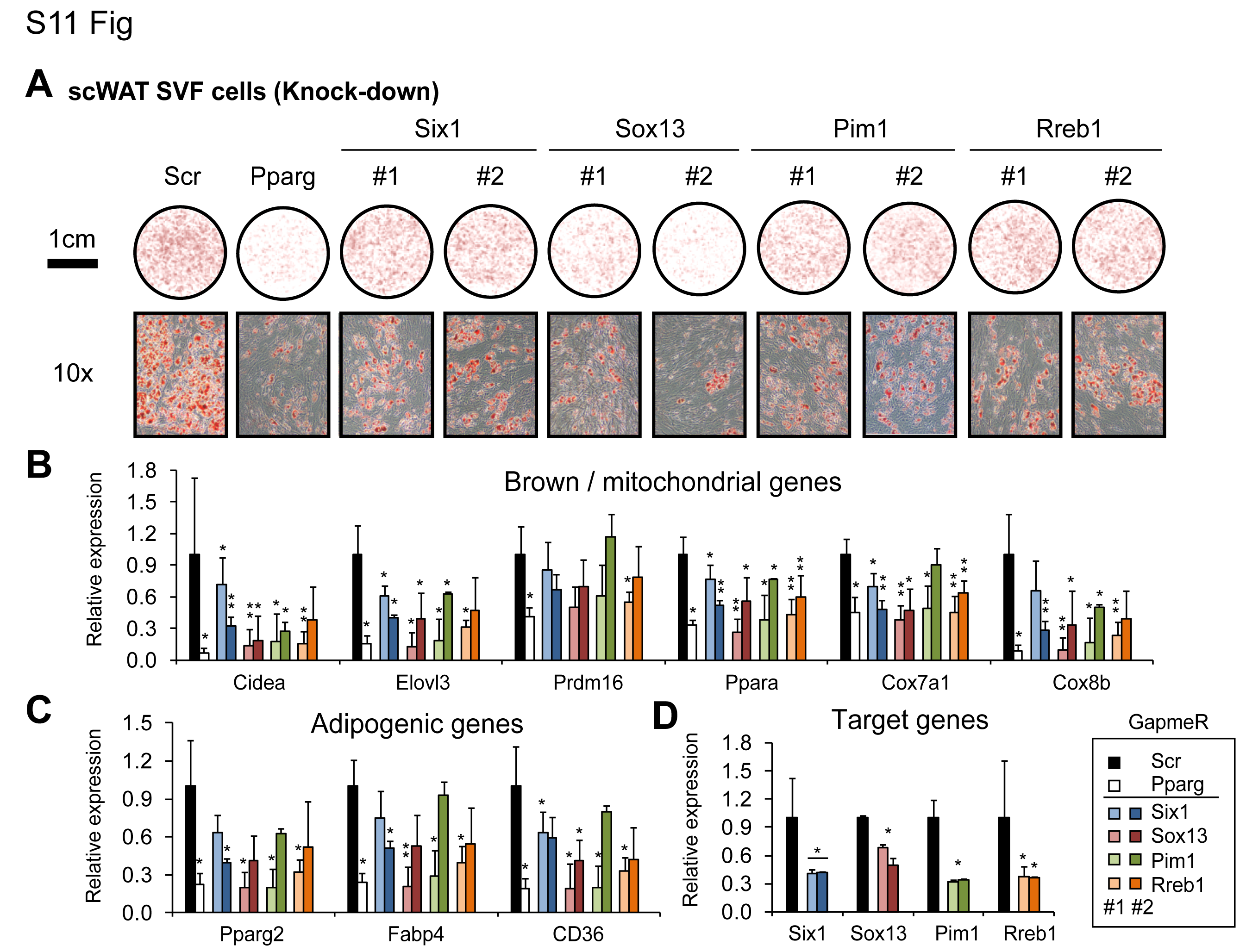

Supplement: S11 Fig — Related to Fig 6. (A) Oil-Red-O staining of SVF cells isolated from scWAT after in vitro differentiation for 7 days. Before adipogenic induction, cells were transfected with two independent (#1 and #2) locked nucleic acid (LNA) longRNA GapmeRs targeting the Six1, Sox13, Pim1, or Rreb1 genes. Scrambled (Scr) or Pparg-targeting LNA GapmeRs served as negative and positive controls, respectively. (B) Expression of brown / mitochondrial marker genes was measured by qRT-PCR on day 7 of adipogenesis. (C) Expression of general adipogenic genes on day 7 of adipogenesis. (D) mRNA levels of targeted genes were assayed 24-hour post transfection. Panels (B)-(D) summarize the average of three independent experiments. Error bars represent standard deviation. p-values (paired student’s t-test): * <0.05; ** <0.01. (TIF) [file pgen.1006474.s011.tif]

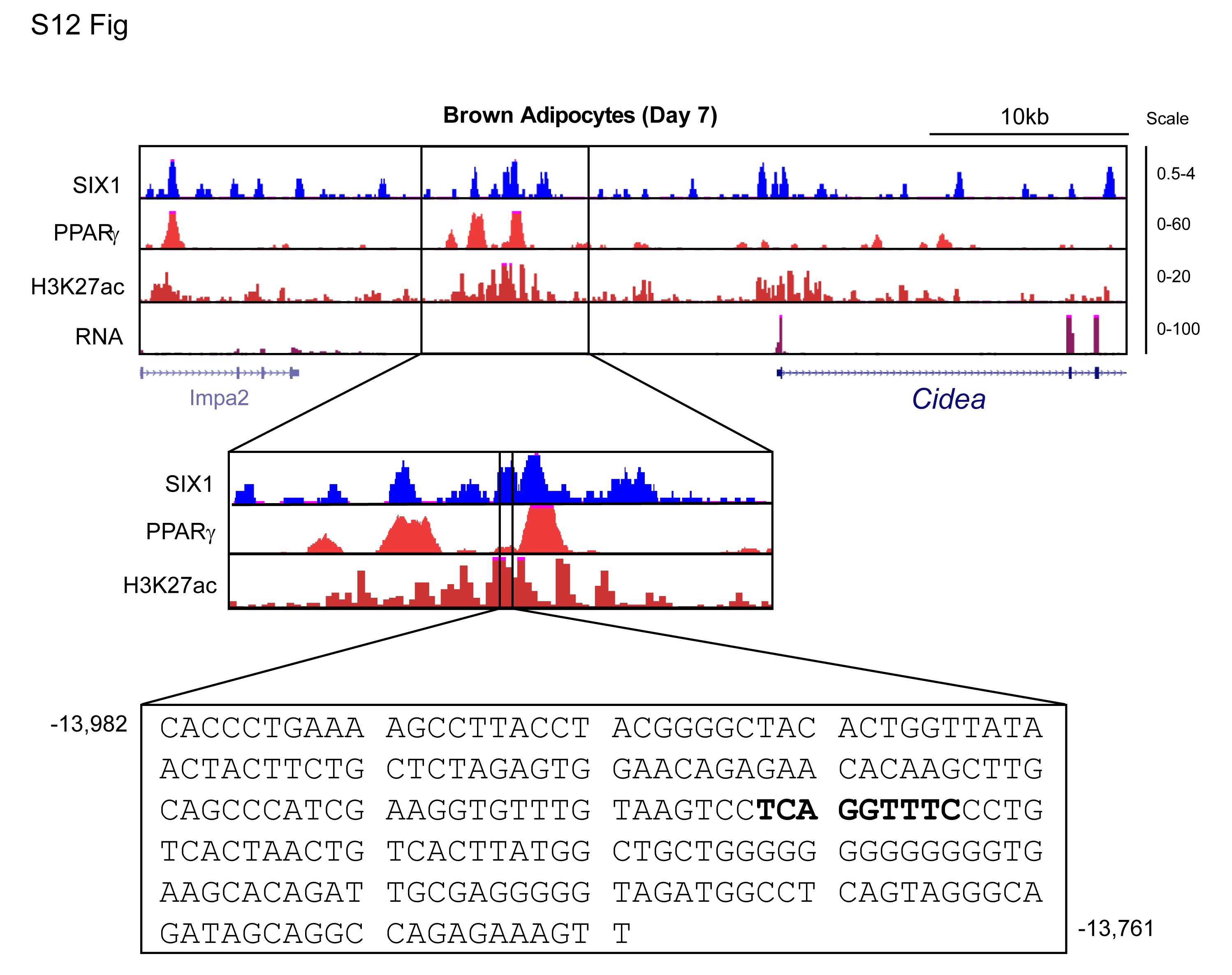

Supplement: S12 Fig — Related to Fig 7. Enlarged view of the upstream region of the Cidea gene reveals the existence of an enhancer element bound by SIX1, PPARγ and marked by H3K27ac. A 222bp SIX1-bound region was PCR-amplified, cloned and tested for its regulatory potential using luciferase assay. SIX1 binding motif (TCAGGTTTC) was highlighted in bold letters. (TIF) [file pgen.1006474.s012.tif]
